# Timely neurogenesis drives the transition from nematic to crystalline nuclear packing during retinal morphogenesis

**DOI:** 10.1126/sciadv.adu6843

**Published:** 2025-05-09

**Authors:** Lucrezia C. Ferme, Allyson Q. Ryan, Robert Haase, Carl D. Modes, Caren Norden

**Affiliations:** ^1^ Gulbenkian Institute for Molecular Medicine, rua da Quinta Grande 6, 2780-156 Oeiras, Portugal (formerly Instituto Gulbenkian de Ciência, IGC).; ^2^ Max-Planck Institute for Molecular Cell Biology and Genetics, MPI-CBG, Pfotenhauerstrasse 108, 01307 Dresden, Germany.; ^3^ Excellence Cluster, Physics of Life, Technische Universität Dresden, Arnoldstrasse 18, 01307 Dresden, Germany.; ^4^ Center for Systems Biology, Pfotenhauerstrasse 108, 01307 Dresden, Germany.; ^5^ Data Science Center, Leipzig University, Humboldtstraße 25, 04105 Leipzig, Germany.; ^6^ Center for Scalable Data Analytics and Artificial Intelligence (ScaDS.AI), Dresden/Leipzig, Germany.

## Abstract

Correct organogenesis depends on the timely coordination of developmental processes, such as cell proliferation, differentiation, and migration. This coordination is particularly critical in crowded tissues, such as pseudostratified epithelia (PSE) that are often found as organ precursors. They are composed of elongated epithelial cells with densely packed nuclei aligned along the apicobasal axis. While cell cycle–dependent nuclear movements in PSE are well studied, less is known about how nuclear packing influences tissue morphogenesis. To investigate this, we analyzed nuclear shapes, sizes, and neighborhood statistics in zebrafish neuroepithelia, focusing on the retinal PSE. We found that nuclei exhibit elongated shapes and biaxial nematic-like orientational order but remain positionally disordered. During retinal development, nuclear packing density increases, approaching theoretical limits. This occurs when the tissue transitions to a laminated structure and nuclear shapes are remodeled. Timely neurogenesis is critical as failure to initiate neurogenesis leads to tissue deformations. These findings highlight the influence of nuclear shape and positioning for organ morphogenesis.

## Teaser

Arrangement of nuclei in densely packed neuroepithelia affects tissue mechanical properties and, consequently, morphogenesis.

## INTRODUCTION

Cell proliferation, migration, and differentiation need to be tightly coordinated in space and time as well as across scales to ensure that functional organs are formed reproducibly during development. This coordination is essential in organismal morphogenesis including the generation of the vertebrate central nervous system (CNS), consisting of spinal cord, retina, and brain. All CNS structures arise from pseudostratified epithelia (PSE) (*1*–*7*), as do brain and retinal organoids (*8*–*10*). PSE also serve as organ precursors in other developmental contexts both in invertebrates and vertebrates, such as lungs and liver in mouse, wing imaginal discs in *Drosophila*, or the embryonic ectoderm in *Nematostella* (*11*–*15*). Thus, PSE are conserved organ precursors across evolution. In general, PSE are highly proliferative tissues featuring a monolayer of apicobasally elongated cells that are attached to a basal lamina (*13*, *16*). Nuclei within these epithelial cells occupy positions all along the apicobasal cell axis during most of the cell cycle, except for during mitosis, which always occurs apically (*1*, *17*–*20*). PSE show high proliferative rates during development and become therefore more and more densely packed with nuclei (*1*, *3*, *5*, *12*, *20*–*22*). This broad occurrence of PSE in diverse developmental contexts and the conservation of these features suggest that pseudostratification can aid proper organogenesis. However, how exactly pseudostratification could positively influence neural and other tissue development is not yet fully understood.

This knowledge gap arises from the fact that so far studies have mainly focused on characterizing cell shape changes and nuclear movements in PSE and how these events affect development (*15*, *22*–*26*). However, the dense nuclear packing that is one of the most distinguishing features of PSE and its possible effects have not been as thoroughly explored. For example, little is known regarding the effects of dense packing of nuclei on PSE morphogenesis and whether pseudostratification could have a role for further tissue development. Based on the fact that nuclei occupy a substantial fraction of the cell volume and considering that nuclear mechanical properties vary depending on cell type (*27*–*30*), recent studies proposed that nuclear positioning and packing could drive mechanical changes in neuroepithelia of the vertebrate retina and of the inner ear (*31*, *32*). It was shown that stalling of nuclei close to the apical side of the proliferating medial epithelial layer is correlated with bending of the mouse cochlear duct, indicating that nuclear positioning can influence tissue shape (*31*). Another study theoretically modeled epithelial tissues and showed that cells become more constrained and progressively more ordered when nuclear-to-cytoplasmic ratios decrease and nuclear volume fractions approximate limiting packing fractions (*32*). Comparisons of the theoretical insights with quantitation of nuclei in optical slices of the densely crowded zebrafish retina underlined the hypothesis that nuclear packing could drive a jamming transition of the tissue (*32*). While being insightful, these studies were either limited to two-dimensional (2D) analyses or dealt with very low sample sizes. Further, they did not experimentally address the direct implications that approaching a maximum nuclear packing density could have for neuroepithelial development. As it was recently shown that epithelial cells in PSE feature complex 3D shapes and numerous neighbor exchanges along their apicobasal axis (*15*, *33*), a 3D appreciation of nuclear packing in neuroepithelia is crucial to understand these tissue types. This analysis is still lacking, however, due to the considerable technical challenges that comes with producing accurate segmentation and analysis of nuclei in volumetric imaging datasets of crowded tissues.

Here, we addressed these challenges using StarDist-3D (*34*) and quantified nuclear packing densities within proliferating neuroepithelia. We focused on the developing zebrafish retina and analyzed the transition from PSE to ordered laminated neuronal structures. During retinal development, multipotent progenitor cells in the PSE proliferate to sustain tissue growth while concomitantly starting to generate neurons (*21*, *35*). These neurons, upon birth at the apical surface, relocate within the retina to the positions at which they later function (*36*–*39*). The emergence of neuronal layers ensures proper connectivity and functionality of the neural tissue (*40*, *41*). When this spatial arrangement of the neurons is impaired, mature tissues can become pathological and dysfunctional (*42*, *43*). While many studies have shed light on how these intracellular mechanisms of proliferation, differentiation and migration are genetically encoded and regulated (*39*, *44*, *45*), less is known about the influence that changes of nuclear packing have on the morphogenesis of the retina. It is also not clear whether and to what extent nuclei are arranged in an ordered or disordered fashion in the retinal PSE and how more ordered tissue structures arise during the development from the seemingly less ordered PSE into a layered neuronal architecture. We here applied concepts from soft matter and solid state physics that are increasingly being adopted to assess packing and order in biology (*46*, *47*). In this context, we compare the arrangement of nuclei in developing neuroepithelia to the arrangement of anisotropic particles in liquid crystals, where the nematic order represents the simplest order to describe a physical state intermediate to a liquid and a solid, with only partially broken continuous symmetry. These nematic liquid crystals feature anisotropic objects that are aligned, on average, along a common orientation axis and thus exhibit orientational order, although they lack positional order (fig. S1), as seen, for example, in actin filament alignment in the cytoskeleton or cell elongation patterns in epithelial monolayers (*47*). Nuclei in the retinal PSE are particularly well suited to the application of these concepts because they are elongated and can fill an appreciable fraction of the total cell space.

Together, our systematic 3D analysis showed that nuclear volume fractions increased continuously over the proliferative phase, reaching theoretical packing fraction limits upon neuronal lamination. We find that nuclei are orientationally ordered and positionally disordered in the proliferating RNE, reminiscent of the nematic-like order prevalent in liquid crystals. This orientational order of nuclei is found to be conserved in a differently shaped neuroepithelium that shows looser packing regimes, the developing hindbrain. Together, this indicates that nematic-like order is a hallmark of pseudostratification. Later in development, neurogenesis progressively remodels nuclear shapes and aids in the transition from the RNE to an even more positionally ordered, crystal-like arrangement of nuclei while maintaining the overall orientational order in the laminated retina. Overall, our results suggest that progressive emergence of nuclear packing order in the RNE enables the close arrangement of newly formed neurons. When this is perturbed, tissue shape is not maintained.

## RESULTS

### Instance 3D segmentation of nuclei in the zebrafish RNE

**Fig. 1.**
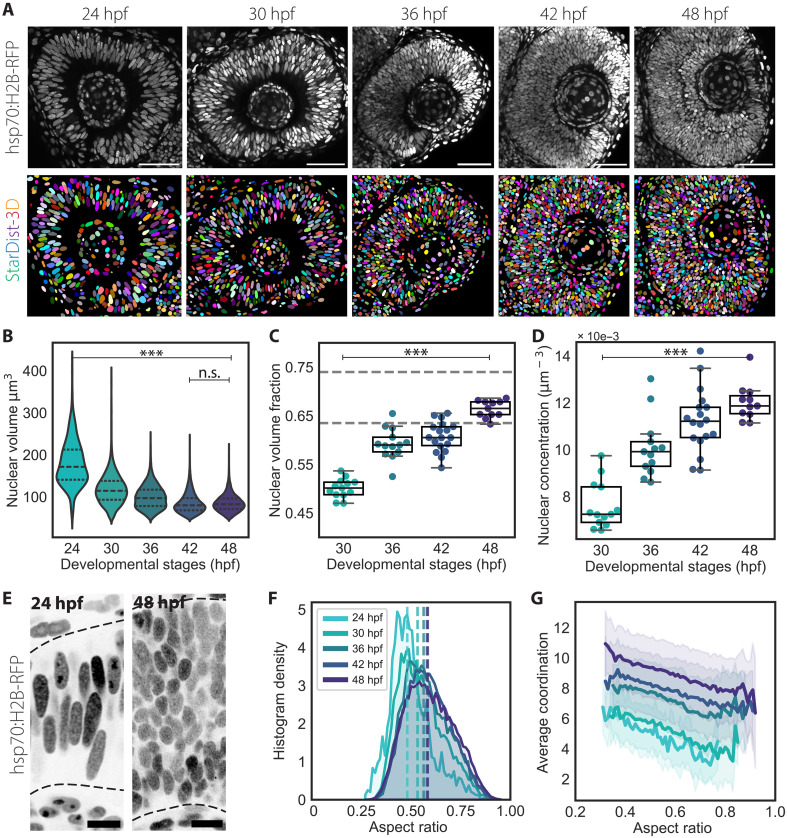
Instance 3D segmentation of nuclei shows increasing nuclear packing densities in the retinal PSE. (**A**) Representative confocal sections of the developing retina between 24 and 48 hpf (top row) and corresponding segmented predictions obtained using StarDist-3D (bottom row). Nuclei are labeled with tg (hsp70:H2B-RFP). Scale bar, 50 μm. (**B**) Distributions of nuclear volumes. *P* values for α = 0.01: *** < 0.0001, results from Mann-Whitney *U* test. n.s. not significant (**C**) Volume fraction occupied by nuclei in ROIs. Segmented lines show the volume fractions φ_s_ ≅ 0.635 and φ_e_ ≅ 0.74 for RCP density of isovolumetric spheres and fully aspherical ellipsoids, respectively. 30 hpf, *N* = 13 embryos; 36 hpf, *N* = 13; 42 hpf, *N* = 18; 48 hpf, *N* = 11. (**D**) Concentration of nuclei found within the ROIs of (B). *P* values for alpha = 0.01: *** < 0.0001 from unpaired two-tailed Student’s *t* test. (**E**) Representative confocal sections of the 24 and 48 hpf retina from (A). Segmented lines mark the apical and basal boundaries of the retina. Scale bar, 10 μm. (**F**) Histogram density distributions of nuclear aspect ratios, which were defined as the ratio between the longer axis length and the mean length between the intermediate and shorter axis. Color-coded segmented lines indicate the mean. (**G**) Correlation between mean number of contacts (average coordination) and nuclear aspect ratios. Solid lines show mean values, and shaded area indicates the SD from the mean. hpf, hours post-fertilization.

To analyze the arrangements of progenitor cell nuclei within the retinal neuroepithelium (RNE) over development, we segmented nuclei using the tg (hsp70:H2B-RFP) zebrafish transgenic line, which marks chromatin and labels all nuclei. The red fluorescent protein (RFP) labeling allowed us to image with lasers at longer wavelengths, reducing light scattering and improving penetration depth. Furthermore, the variable expression of H2B-RFP under a heat shock promoter facilitated more efficient segmentation than what would be possible with more evenly distributed staining, such as DRAQ5, or constructs expressed under a constitutive promoter (fig. S2, A and B). Tg (hsp70:H2B-RFP) embryos were staged and fixed starting at 24 hours post-fertilization (hpf), when the optic cup consists of only proliferating progenitors, every 6 ± 1 hours until 48 hpf, by which time neuronal lamination is ongoing (Fig. 1A).

Locating and separating individual nuclei within a 3D volume is a computer vision task, called instance segmentation, which assigns a label mask to each single object. We annotated 14 cropped 3D images with approximately 40 to 300 labeled nuclei each and used this dataset to train a StarDist-3D model (*34*), which we then applied for segmentation in the volumetric dataset (fig. S2, C and D, and table S1). Based on this dataset, we established an image analysis pipeline to extract several nuclear shape descriptors (fig. S3A), such as nuclear size and axes’ length (fig. S4, A to C, and movie S1), and explore the arrangement of nuclei within the RNE (figs. S3, B and C and S4, D to I, and movie S2). For further details on these exact analysis pipelines, see Materials and Methods.

### Nuclear packing in the retinal PSE reaches theoretical close packing densities over development, while nuclei keep an ellipsoidal shape

To evaluate nuclear packing, we quantified nuclear size, counts, and the volume fraction that nuclei occupy within defined regions of interests (ROI) in the central part of the RNE (see Materials and Methods and movie S3). We found that nuclear volumes decrease between 24 and 42 hpf, until the onset of neuronal lamination (Fig. 1B, fig. S4A, and Table 1). This decrease was accompanied by a progressive increase of the nuclear concentration, i.e., number of nuclei per volume, and of the nuclear volume fractions between 30 and 48 hpf (Fig. 1, C and D). The median volume fraction $\bar{\varphi }$ reached values up to φ_s_ ≅ 0.635 (Fig. 1C), a value that is generally known as the random close packing (RCP) density of isovolumetric 3D spheres reaching their maximum density for generic disordered packings (*48*). The RCP density varies greatly between 2D and 3D situations, so that the RCP value φ_d_ for monodisperse discs in 2D is higher than for spheres in 3D and falls in the range 0.84 < φ_d_ < 0.89 (*49*), showing the relevance that considering nuclear packing in 3D entails. However, nuclei in the RNE are not simply isovolumetric spheres but more closely resemble ellipsoids (Fig. 1E). Ellipsoidal particles are known to pack more densely than φ_s_, reaching RCP densities in the range 0.64 < φ_e_ < 0.74 (*50*) By 48 hpf, most of the analyzed retinas had volume fraction values higher than φ_s_, in the range of φ_e_, implying that nuclear packing in the retinal PSE was possibly approaching a jamming transition boundary for ellipsoidal particles. Here, the term “jamming transition boundary,” refers to the packing density above which a disordered system becomes dynamically arrested or effectively solidifies. This means that single particles cannot freely move and exchange neighbors because they are confined by the particle-particle interactions of their direct neighbors. This concept differs slightly from the previous definition of RCP density as the distribution of configurations that are available for a “random” packing can subtly depend on the history or preparation of the system (*51*). Nevertheless, for the purposes of this study, we consider them to be effectively equivalent. This is because an appreciable shift to the transition density relative to the RCP value requires a specific time course of preparation that is not expected to occur in the retinal PSE. To test whether nuclear shapes changed due to increased packing, we quantified nuclear shapes over the proliferative phase. Our analysis showed that most nuclei retained their ellipsoidal shape between 24 and 48 hpf and that nuclear volume reduction resulted mainly from a progressive shortening of the major axis until 42 hpf (Fig. 1F; fig. S5, A, E, and F; and Table 1). In contrast, volume distributions of nuclei between 42 and 48 hpf, which correspond to the onset of neuronal lamination, did not change (Fig. 1B). This suggested that nuclear size changes could be affected by nuclear packing and by neuronal lamination.

**Table 1.** Volumes, axes lengths, and aspect ratios of nuclei located in the central RNE and in the hindbrain neuroepithelium. Mean and SD are reported for each developmental stage (hpf). Number of embryos (*N*) and number of nuclei in ROIs (*n*) per stage in the retina: 24 hpf, *N* = 6, *n* = 1942; 30 hpf, *N* = 13, *n* = 7362; 36 hpf, *N* = 13, *n* = 9379; 42 hpf, *N* = 18, *n* = 28193; 48 hpf, *N* = 11 embryos, *n* = 25313 nuclei. Number of embryos (*N*) and number of nuclei in ROIs (*n*) per stage in the hindbrain: 18 hpf, *N* = 20, *n* = 13661; 22 hpf, *N* = 11, *n* = 6682; 24 hpf, *N* = 7, *n* = 6049; 30 hpf, *N* = 6 embryos, *n* = 9126 nuclei. Mann-Whitney tests between previous and following stages were performed both for nuclear volume and for nuclear aspect ratios distributions, i.e., comparisons were made between values from 24 and 30 hpf, between 30 and 36 hpf, between 36 and 42 hpf, and between 42 and 48 hpf. The same procedure was performed for measurements from the hindbrain. *P* values are shown for each test.

		Retinal neuroepithelium		
		Nuclear volume (μm^3^)		
Stage		μ ± σ	H0: same distribution	*P* value
24	hpf	177.87 ± 55.77	–	–
30	hpf	113.52 ± 39.92	(24 hpf) = (30 hpf)	*P* ≪ 0.0001
36	hpf	95.76 ± 30.54	(30 hpf) = (36 hpf)	*P* ≪ 0.0001
42	hpf	81.67 ± 23.40	(36 hpf) = (42 hpf)	*P* ≪ 0.0001
48	hpf	81.92 ± 23.06	(42 hpf) = (48 hpf)	0.1798
		Nuclear axis lengths (μm)		
Stage		A (μ ± σ)	B (μ ± σ)	C (μ ± σ)
24	hpf	11.5 ± 2.59	6.17 ± 0.87	4.81 ± 0.66
30	hpf	9.08 ± 2.00	5.50 ± 0.9	4.31 ± 0.70
36	hpf	8.34 ± 1.68	5.25 ± 0.78	4.22 ± 0.57
42	hpf	7.85 ± 1.43	5.00 ± 0.71	4.04 ± 0.57
48	hpf	7.85 ± 1.33	5.02 ± 0.72	4.08 ± 0.58
		Aspect ratio [A/ (B + C)]		
Stage		(μ ± σ)	H0: same distribution	*P* value
24	hpf	0.49 ± 0.101	–	–
30	hpf	0.55 ± 0.111	(24 hpf) = (30 hpf)	*P* ≪ 0.0001
36	hpf	0.58 ± 0.113	(30 hpf) = (36 hpf)	*P* ≪ 0.0001
42	hpf	0.59 ± 0.111	(36 hpf) = (42 hpf)	0.016
48	hpf	0.59 ± 0.120	(42 hpf) = (48 hpf)	0.899
		Hindbrain neuroepithelium		
		Nuclear volume (μm^3^)		
Stage		μ ± σ	H0: same distribution	*P* value
18	hpf	315.68 ± 41.88	–	–
22	hpf	279.37 ± 46.43	(18 hpf) = (22 hpf)	0.2323
24	hpf	241.93 ± 75.72	(22 hpf) = (24 hpf)	*P* ≪ 0.0001
30	hpf	201.66 ± 20.11	(24 hpf) = (30 hpf)	*P* ≪ 0.0001
		Nuclear axis lengths (μm)		
Stage		A (μ ± σ)	B (μ ± σ)	C (μ ± σ)
18	hpf	9.34 ± 1.82	6.57 ± 0.77	5.63 ± 0.70
22	hpf	9.68 ± 1.80	6.48 ± 0.77	5.45 ± 0.66
24	hpf	8.99 ± 1.61	6.09 ± 0.68	5.01 ± 0.64
30	hpf	7.97 ± 1.49	5.79 ± 0.81	4.87 ± 0.61
		Aspect ratio [A/ (B + C)]		
Stage		(μ ± σ)	H0: same distribution	*P* value
18	hpf	0.67 ± 0.13	–	–
22	hpf	0.63 ± 0.13	(18 hpf) = (22 hpf)	*P* ≪ 0.0001
24	hpf	0.64 ± 0.13	(22 hpf) = (24 hpf)	0.292
30	hpf	0.69 ± 0.10	(24 hpf) = (30 hpf)	*P* ≪ 0.0001

To explore the relation between elongated ellipsoidal morphology and RCP densities, we compared the number of nearby neighbors per nucleus to their aspect ratio. Because neighboring nuclei do not physically touch due to the presence of cell membranes between them, they were defined as those nuclei that are in contact after a small dilation of each nucleus’ outline boundaries, here referred to as touching neighbors (fig. S4, D and E). After testing different radii of dilation, we adopted a radius of 0.25 μm as minimal effective touching–through–cell membrane length scale, since this number was the minimal step size of voxels in our imaging dataset. This analysis confirmed that the average contact number Z and the nuclear aspect ratio of each nucleus were linearly correlated, meaning that more elongated nuclei tend to contact a higher number of neighbors, referred to as the average coordination number (Fig. 1G). This correlation between average coordination and aspect ratio did not vary across stages, while the range of average coordination increased, reaching contact number values closer to 10 by 48 hpf. The maximum contact numbers for ordered arrangements of isovolumetric spheres and ellipsoids are known to be at Z_s_ = 12 and Z_e_ = 14, respectively (*50*). Thus, nuclei in the RNE approach limiting RCP densities at the onset of neuronal lamination.

Overall, the quantification of nuclear shape descriptors from 3D instance segmentation enabled us to follow nuclear packing changes in the RNE over development. Our results indicate that nuclear shape and size in progenitor cells determine nuclear packing densities in the proliferating RNE and allow the tissue to reach high nuclear volume fractions, without impeding progenitor nuclear movements (*35*, *52*). For details on image analysis code and the trained StarDist-3D model, see Materials and Methods.

### Nuclei are arranged in a nematic-like order in the proliferative phase

The increase in nuclear volume fractions and contact numbers suggested that nuclei approach a closer packing density during the proliferative phase. We thus asked whether the emergence of positional order, defined as regular spacing of nuclei in the tissue, could enable more cells to be accommodated within the tissue without reaching its maximum packing capacity. To test this idea, we analyzed whether nuclear packing varied spatially within the retinal PSE and/or whether nuclei showed positional order during the proliferative phase.

**Fig. 2.**
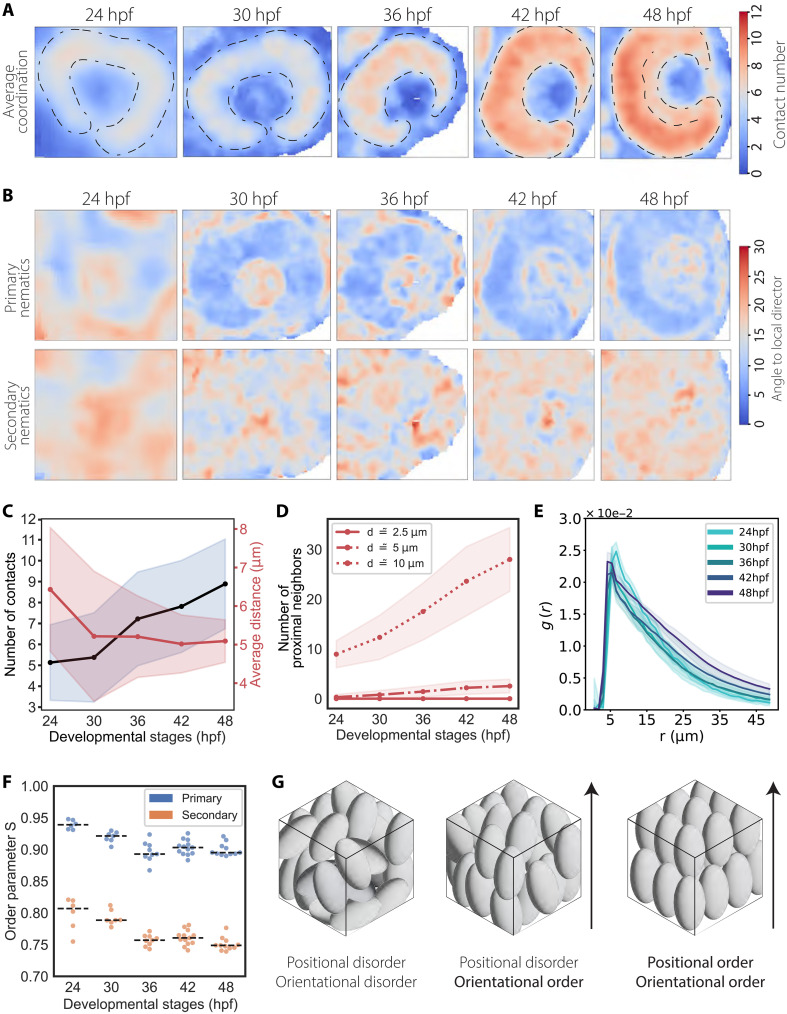
Nuclei are positionally disordered but orientationally ordered in the retinal PSE. (**A**) Median intensity projections of representative smoothened imaging datasets reporting the average coordination number for nuclei within a given 3D window size of 10 μm by 10 μm by 10 μm. The representative images shown are the same as in Fig. 1A. Outline of the retinal PSE is marked by segmented line, together with the RGL at 48 hpf. (**B**) Median intensity projection of representative smoothed imaging datasets reporting the mean angle of misalignment between primary nematic axes and the local primary nematic direction (top row) and the mean angle of misalignment between secondary nematic axes and the local secondary nematic direction (bottom row). Measurements were collected and averaged within a given 3D window size of 10 μm by 10 μm by 10 μm (top row). (**C**) Mean number of contacts within ROIs increases, while the average distance between touching neighbours stays constant. Solid lines correspond to mean values, and shaded area indicates the SD from the mean. (**D**) Mean number of proximal neighbors per nucleus within different distance radii. Solid lines correspond to mean values, and shaded area indicates the SD from the mean. (**E**) RDF *g* (*r*) calculated for nuclei in ROIs at different developmental stages between 24 and 48 hpf indicate a liquid-like state. Solid lines correspond to mean values, and shaded area indicates the SD from the mean. (**F**) Graphs for order parameter S for primary and secondary nematics S over development. (**G**) Examples of monodisperse ellipsoids arranged from fully disordered to fully ordered packing.

A method to evaluate the nuclear packing environment is to analyze the average coordination number of nuclei across the apicobasal axis of the tissue. To do this, we performed image smoothing by averaging our measurements across the voxels of our segmentation dataset and visually inspected the result (fig. S3, B and C). We found that the average coordination of nuclei in the PSE increased continuously between 24 and 48 hpf, reaching higher values in the inner part of the tissue (Fig. 2A). The average contact numbers were lower in the retinal ganglion cell layer (RGL), which formed between 42 and 48 hpf, suggesting that neuronal lamination reduced nuclear packing densities locally (Fig. 2A). To discriminate between a scenario in which random nuclear packing is simply increasing and a scenario in which the positional arrangement of nuclei is changing at the same time, we analyzed the mean internuclear centroid-to-centroid distance of touching neighbors. We found that this distance initially decreased but plateaued at around 5 μm between 30 and 48 hpf, while the mean contact numbers continued to increase (Fig. 2C). This minimal internuclear distance was confirmed by the analysis of proximal neighbors within a set radius, which showed that the number of these neighbors rose in the range of 5- and 10-μm radii (Fig. 2D). These results reinforced the previous observations that nuclear packing in the developing tissue becomes denser between 30 and 48 hpf. Next, we explored whether any periodic positional ordering of nuclei emerged over development by computing the radial distribution function (RDF). This function describes how particle density changes as a function of distance between particles. Consequently, the RDF provides insights on the positional order of particles, here nuclei. Nuclei in a solid-like structure would be found at regular distances and would therefore be represented by the RDF as regularly spaced peaks in the graph, while a liquid-like state would be represented as one peak which declines as the distance increases. In the retinal PSE, we found the highest probability to find nuclei at around 5-μm internuclear distance, matching the minimal internuclear distance observed above and thus associated to nearest neighbors, while the RDF smoothly decreases at longer distances (Fig. 2E). Such a profile in the RDF indicates that between 24 and 48 hpf, no regular spacing of nuclei is detected, meaning that nuclei are positionally disordered as in a liquid-like state.

Note, however, that nuclei feature an anisotropic morphology throughout the proliferative phase and, due to the elongation of neuroepithelial cells, appear to align along the apicobasal cell axis. Depending on the extent of this alignment and given the positional disorder shown by the RDF, anisotropic nuclei could be arranged in a nematic-like order similar to liquid crystals. This means that they are positionally disordered but orientationally ordered. This would allow nuclei to reach a more rigid configuration in which particles can nevertheless still flow as if in a liquid-like phase. This collective arrangement of nuclei could potentially explain how neuroepithelial cells in proliferating PSE reach high nuclear packing densities without impairing movements along the apicobasal cell axis. To understand if nuclei in the RNE were organized in this manner and to monitor their orientation during the proliferative phase, we calculated the primary nematic axis of each nucleus (fig. S4, G and H, and movie S2) and their misalignment angle to the average angle of surrounding nuclei from our imaging dataset. We found that nuclei were strongly aligned throughout development, with an average misalignment angle of only around 10° (Fig. 2B). Computing the local orientation order parameter further confirmed the presence of strong alignment (Fig. 2F). The same analysis was performed for a secondary nematic axis, which was defined as the elongation axis on the midplane normal to the primary nematic (fig. S4, G and I, and movie S2). This analysis also revealed the presence of a consistent alignment, although weaker than that along the primary axis (Fig. 2, B and F). This secondary alignment suggests that nuclei in the RNE are actually organized as a biaxial nematic.

Overall, our analysis demonstrated that packing densities vary spatially within the RNE and that nuclei are positionally disordered as in a liquid-like arrangement but present 1° of order as in a more solid-like configuration (Fig. 2G). This suggests that nuclei in the RNE are arranged with biaxial nematic order, similar to that found in many liquid crystals.

### Nematic-like order is a hallmark of pseudostratification also at looser packing regimes

**Fig. 3.**
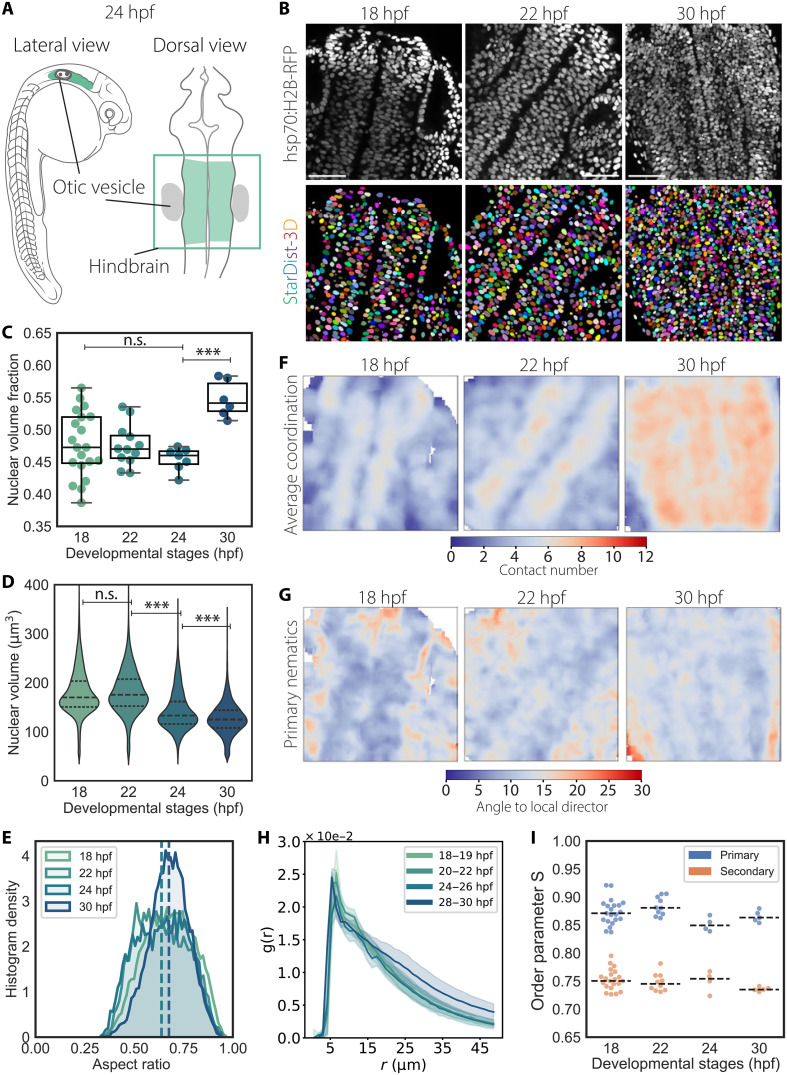
Instance 3D segmentation of hindbrain PSE reveals nematic ordering of nuclei at lower packing regimes. (**A**) Scheme of a 24 hpf of zebrafish marking the region of the hindbrain that was imaged and quantified. (**B**) Representative images of the hindbrain PSE during early development (top row) and corresponding instance segmentation using StarDist-3D model (bottom row). Nuclei are labeled with Tg (hsp70:H2B-RFP). Scale bar, 50 μm. (**C**) Nuclear volume fractions over time. 18 hpf, *N* = 20 embryos; 22 hpf, *N* = 11; 24 hpf, *N* = 7; 30 hpf, *N* = 6. *P* values for alpha = 0.01: *** < 0.0001 from unpaired two-tailed Student’s *t* test. (**D**) Violin plot of nuclear volume distributions over time. Segmented lines indicate first and third quartiles and medians. *P* values for alpha = 0.01: *** < 0.0001; results from Mann-Whitney *U* test. (**E**) Histogram density distributions of nuclear aspect ratios. Segmented lines indicate the mean. (**F**) Median intensity projections of representative smoothed imaging datasets reporting the average coordination number for nuclei within a window size of 10 μm by 10 μm by 10 μm. The representative images shown are the same as in (B). (**G**) Median intensity projection of representative smoothed imaging datasets reporting the mean angle of misalignment between primary nematic axes and the local primary nematic direction within a window size of 10 μm by 10 μm by 10 μm. The representative images shown are the same as in (B). (**H**) RDF *g* (*r*) between 18 and 30 hpf. Solid lines correspond to mean values, and shaded area indicates the SD from the mean. (**I**) Graph for order parameter S for primary and secondary nematics.

It was previously shown that nuclear positioning mechanisms vary between differently shaped PSE within the same organism, such as the developing zebrafish retina and hindbrain (*18*, *53*). While the retinal PSE shows hemispheric morphology, the developing hindbrain is a straight tubular PSE. We thus asked whether despite these differences in architecture and nuclear translocation mechanisms nuclear packing densities and ordering were conserved or whether they differed between retinal and hindbrain PSE during the proliferative phase and/or at the onset of differentiation. To this end, we carried out the same 3D analysis in the developing hindbrain as done in the retina. Hindbrain neuroepithelia were analyzed at set intervals between 18 and 30 hpf, i.e., between the formation of the neural rod and delamination of newly formed neurons (*54*–*56*), as shown by HuC/HuD staining (Fig. 3, A and B, and fig. S6A). In contrast to the retinal PSE, we found that nuclear volume fraction and nuclear concentration did not increase until neuronal lamination at 30 hpf (Fig. 3C and fig. S6B) while still showing a positive correlation same as in the retinal PSE (fig. S6C). Between 18 and 24 hpf, nuclear packing remained in a looser regime than what was found in the retinal PSE, while nuclear volumes decreased over time starting around 24 hpf at the onset of neuronal lamination (Fig. 3D and Table 1). Nuclei presented aspect ratios closer to 1, indicating a more spherical shape compared to nuclei in the retinal PSE, and nuclear volume reduced isotropically (Fig. 3E; fig. S6, D to F; and Table 1). Nevertheless, nuclear morphologies still inversely correlated with average coordination numbers (fig. S6G). The mean internuclear distance changed marginally between 18 and 30 hpf (fig. S6, H and J), although the average numbers of touching neighbors after dilation and of proximal neighbors within a 10-μm radius substantially increased between 24 hpf and 30 hpf (Fig. 3F and fig. S6, I and J). Moreover, nuclei in the hindbrain were arranged with nematic order as seen in the RDF analysis and the alignment of the primary nematic axes (Fig. 3, G to I). Together, this suggests that the arrangement of nuclei with nematic order is conserved across different PSE, regardless of their packing density or tissue shape.

### Neurogenesis coincides with nuclear shape remodeling and leads to aligned crystal-like arrangements within the laminating retina

Our analysis of nuclear packing in the RNE over development showed that nuclear volume fractions increase during the proliferative phase and reach the theoretical limiting density φ_s_ at the onset of neuronal lamination. In addition, we observed spatial changes in nuclear arrangements upon neurogenesis and during the formation of the RGL (Fig. 2A). Because cell fate specification entails cell shape changes and repositioning within the tissue, we compared nuclear shape distributions and arrangements between the different neuronal layers: the RGL, the outer nuclear layer (ONL) hosting photoreceptor cells, and the inner nuclear layer (INL), hosting horizontal, bipolar, and amacrine cells that is located in between the RGL and ONL. In all layers, we assessed how nuclear packing changes during the transition from the retinal PSE to the laminated retina.

**Fig. 4.**
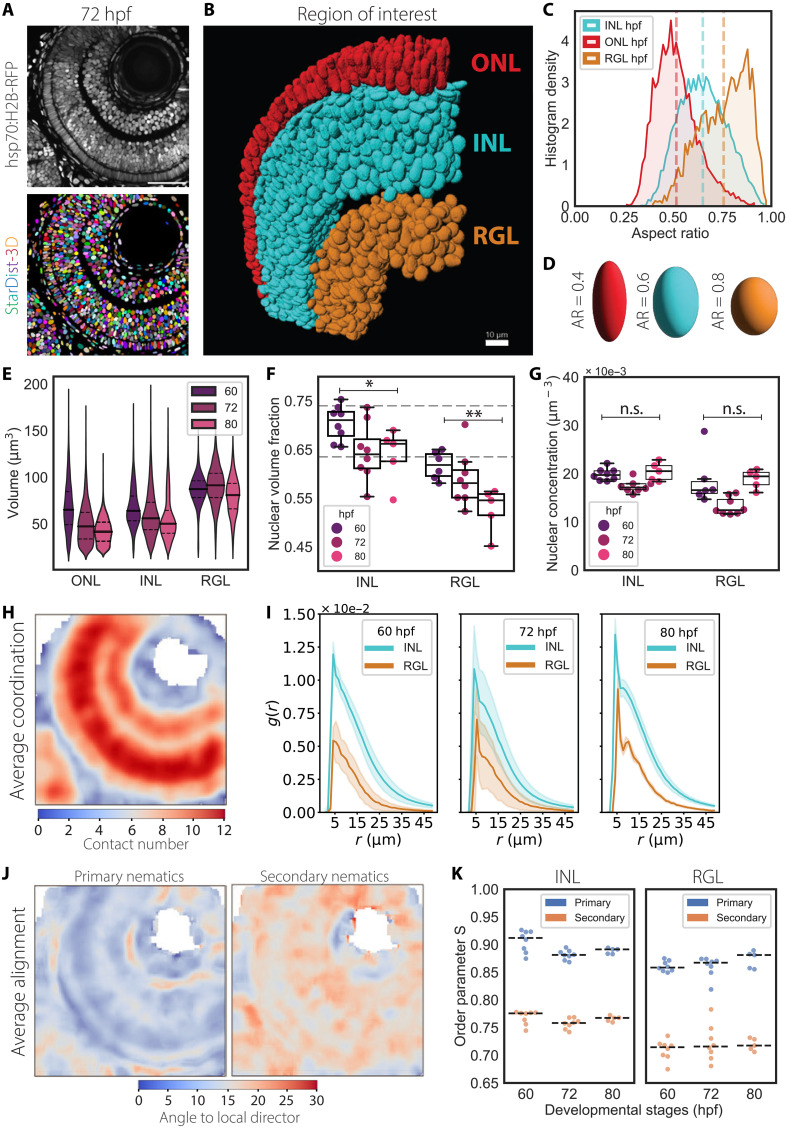
Nuclear packing reaches theoretical limiting density as nuclear shapes and sizes are remodeled during neuronal lamination. (**A**) Representative images of the laminated retina at 72 hpf (top) and corresponding instance segmentation using StarDist-3D model (bottom). Nuclei are labeled with Tg (hsp70:H2BRFP). Scale bar, 50 μm. (**B**) 3D-rendering of 72 hpf of ROI from (A) and color-coded for distinct nuclear layers. Scale bar, 10 μm. (**C**) Histogram density distribution for nuclear aspect ratios at 72 hpf. Segmented lines indicate the mean values for each distribution. (**D**) Schematics of prolate ellipsoids having aspect ratios as shown in (C). (**E**) Nuclear volume fractions of nuclei in the INL and RGL. 60 hpf, *N* = 7; 72 hpf, *N* = 8; 80 hpf, *N* = 5 embryos. (**F**) Concentration of nuclei found within the ROIs of (E). *P* values for alpha = 0.01: 0.01 < * < 0.05 and ** < 0.01 from unpaired two-tailed Student’s *t* test. (**G**) Nuclear volume distributions. See Table 2 for test statistics. (**H**) Median intensity projections of the image in (A) reporting the average coordination number for nuclei within a window size of 10 μm by 10 μm by 10 μm. (**I**) RDF *g* (*r*) in INL and RGL overdevelopment. Solid lines correspond to mean values, and shaded area indicates the SD from the mean. (**J**) Median intensity projections of the image in (A) reporting the local angle of misalignment for the primary (left) and secondary (right) nematic axes within a window size of 10 μm by 10 μm by 10 μm. (**K**) Graphs for order parameter S for primary and secondary nematics.

To quantitatively track changing nuclear morphologies of emerging neurons, we trained a different StarDist-3D model on a manually annotated dataset that specifically included annotations from the nuclear layers of laminated retinas between 48 hpf, by which time neuronal lamination has started, and 80 hpf, when lamination is complete (Fig. 4, A and B, and fig. S1E). We found that nuclear aspect ratios were differently distributed across the three layers (Fig. 4C), especially along the shorter axis (fig. S5, C and I), while the distribution of the major axis length did not significantly vary across the three layers and progressively decreased over development (fig. S5, C and H, and Table 2). As a result, nuclei in the RGL and INL became more spherical, while photoreceptor precursors in the ONL featured an elongated rod-like shape (Fig. 4, C and D). Across all three layers nuclear volumes progressively decreased with time (Fig. 4E and Table 2). As nuclei assumed different shapes and sizes depending on their neuronal fate, the linear correlation between number of contacts and nuclear aspect ratios was lost across all three layers compared to nuclei in the RNE (fig S5G and Table 2). This, together with the fact that the mean internuclear distance did not show major changes during neuronal lamination (fig. S5J), suggested that nuclei had reached their closest packing configuration by 80 hpf.

**Table 2.** Volumes, axes lengths, and aspect ratios of nuclei located in the central part of the laminated retina. Mean and SD are reported for each layer (ONL, INL, and RGL) and developmental stage (hpf). Number of embryos (*N*) per stage: 60 hpf, *N* = 7; 72 hpf, *N* = 8; 80 hpf, *N* = 5 embryos. Number of nuclei in ROIs (*n*) per stage and layer: ONL at 60 hpf, *n* = 4270; ONL at 72 hpf, *n* = 4006; ONL at 80 hpf, *n* = 3313; INL at 60 hpf, *n* = 10684; INL at 72 hpf, *n* = 11028; INL at 80 hpf, *n* = 7715; RGL at 60 hpf, *n* = 2928; RGL at 72 hpf, *n* = 3271; RGL at 80 hpf, *n* = 1983. Mann-Whitney tests between previous and following developmental stages were performed both for nuclear volume and for nuclear aspect ratios distributions within each layer, e.g., comparisons were made between values from 60 and 72 hpf and between 72 and 80 hpf for nuclei located in the INL. *P* values are shown for each test.

			Laminated retina				
			Nuclear volume (μm^3^)				
Layer	Stage		μ ± σ	H0: same	distribution		*P* value
ONL	60	hpf	56.77 ± 29.12		–		–
	72	hpf	45.89 ± 22.80	(60 hpf)	=	(72 hpf)	*P* ≪ 0.0001
	80	hpf	36.80 ± 16.56	(72 hpf)	=	(80 hpf)	*P* < 0.001
INL	60	hpf	59.11 ± 23.33		–		–
	72	hpf	58.39 ± 24.37	(60 hpf)	=	(72 hpf)	*P* ≪ 0.0001
	80	hpf	53.46 ± 24.45	(72 hpf)	=	(80 hpf)	*P* < 0.001
RGL	60	hpf	79.41 ± 19.44		–		–
	72	hpf	90.11 ± 21.24	(60 hpf)	=	(72 hpf)	0.013
	80	hpf	79.58 ± 20.19	(72 hpf)	=	(80 hpf)	*P* ≪ 0.0001
			Nuclear axis lengths (μm)				
Layer stage			A (μ ± σ) B (μ ± σ) C (μ ± σ)				
ONL	60	hpf	7.11 ± 1.60	4.28	±	0.99	3.41 ± 0.86
	72	hpf	7.02 ± 1.53	3.94	±	0.80	3.12 ± 0.66
	80	hpf	6.80 ± 1.39	3.54	±	0.62	2.89 ± 0.55
INL	60	hpf	6.89 ± 1.29	4.44	±	0.80	3.63 ± 0.70
	72	hpf	6.47 ± 1.09	4.55	±	0.76	3.73 ± 0.73
	80	hpf	5.98 ± 1.05	4.44	±	0.73	3.70 ± 0.72
RGL	60	hpf	6.82 ± 1.02	5.15	±	0.59	4.32 ± 0.59
	72	hpf	6.80 ± 1.00	5.44	±	0.54	4.70 ± 0.59
	80	hpf	6.38 ± 0.87	5.18	±	0.51	4.57 ± 0.57
			Aspect ratio [A/ (B + C)]				
Layer Stage			(μ ± σ) H0: same distribution *P* value				
ONL	60	hpf	0.55 ± 0.13		–		–
	72	hpf	0.50 ± 0.10	(60 hpf)	=	(72 hpf)	*P* < 0.001
	80	hpf	0.48 ± 0.09	(72 hpf)	=	(80 hpf)	*P* < 0.001
INL	60	hpf	0.59 ± 0.11		–		–
	72	hpf	0.65 ± 0.11	(60 hpf)	=	(72 hpf)	*P* ≪ 0.0001
	80	hpf	0.69 ± 0.11	(72 hpf)	=	(80 hpf)	*P* < 0.001
RGL	60	hpf	0.70 ± 0.11		–		–
	72	hpf	0.75 ± 0.10	(60 hpf)	=	(72 hpf)	0.011
	80	hpf	0.77 ± 0.10	(72 hpf)	=	(80 hpf)	0.1

To explore how the local packing environment changed over lamination, we performed the same analysis of nuclear packing order as done for the proliferative phase. Because photoreceptors in the ONL are organized in a contiguous monolayer at the tissue boundaries of the retina and are visibly arranged in a regular linear pattern (Fig. 4B), we focused our analysis on the INL and the RGL. In the INL, nuclei reached their highest packing densities at around 60 hpf (Fig. 4F), with values approximating φ_e_ ≅ 0.74. This corresponds to the densest possible crystalline packing of spheres, in an face centered cubic (FCC) crystal; it is also known to be the RCP density for fully aspherical ellipsoids (*50*). This means that, at this stage, nuclei in the INL are possibly approaching a theoretical nuclear crystallization (or jamming) transition boundary. Between 60 and 72 hpf, both nuclear packing and nuclear concentration decreased in the INL and RGL, possibly as a consequence of tissue growth and positional ordering (Fig. 4, F and G). Correlations between volume fractions and nuclear concentration differed when the INL or RGL results were compared to the retinal PSE (fig. S5, B and D), suggesting a distinct proliferation and differentiation state of cells in these layers, where retinal ganglion cells mature earlier than other neurons (*36*, *39*). Visual inspection of smoothened images confirmed that nuclei in the delaminating retina reached higher contact numbers compared to the RNE at 48 hpf, with the INL featuring the closest configuration (Fig. 4H). When calculating the RDF of nuclei in the INL and RGL to determine whether nuclear arrangements were shifting to more regular structures during neuronal lamination, we found discrete peaks in the RDF by 80 hpf both in the INL and in the RGL. This indicated the highest probability to find a nucleus around 5- and 10-μm radii (Fig. 4I), suggesting that positionally ordered, periodic arrangements of nuclei emerge during neuronal lamination. This is different to the RNE where only orientational order was observed. When we analyzed the alignment of nuclei across the three neuronal layers, we found that nuclei maintained a strong orientation along their primary nematic axis even at later stages of development (Fig. 4, J and K).

We conclude that differentiation of neuronal precursors coincides with changes in nuclear shape, alongside the formation of a more orientationally and positionally ordered, crystal-like arrangement of nuclei in the INL and RGL. In the ONL, each photoreceptor is positioned adjacent to its neighbor to form a densely packed monolayer of elongated nuclei arranged in a regular pattern (*57*). Despite changes in morphology and position, nuclei of retinal neurons remain aligned along the radial axis of the neural retina. This was similar to the alignment seen in the proliferative RNE. This shows that orientational order is established early on in development and is conserved in the laminated retina during the emergence of a crystalline positional order of nuclei.

### Modelled material properties of the developing RNE switch between a cellular-dominated and nuclear-dominated states, affecting tissue shape

**Fig. 5.**
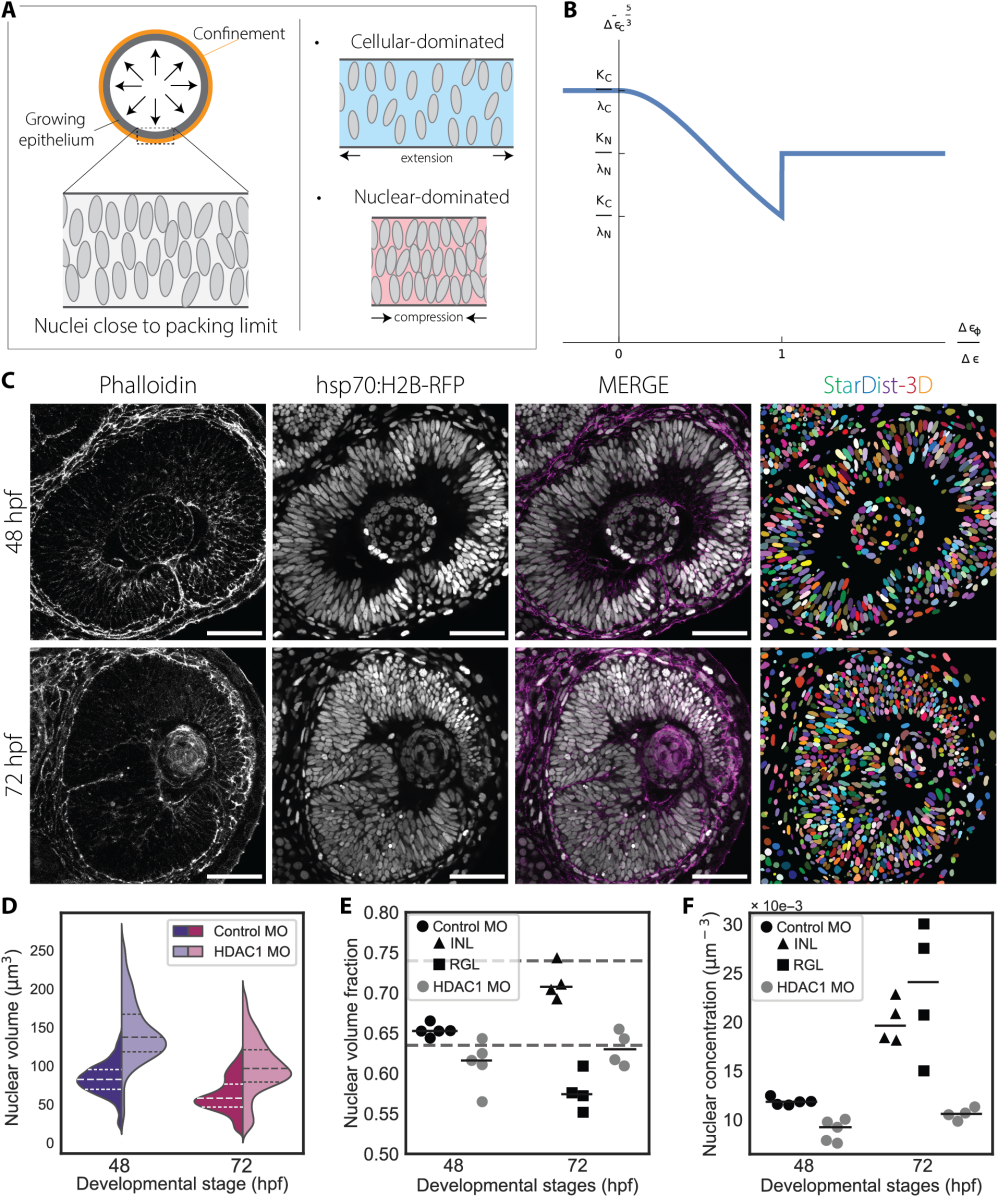
Nuclear-dominated mechanical state leads to buckling when delamination does not occur. (**A**) Theoretical model of a growing epithelium as an elastic band under confinement. Near the nuclear jamming threshold, tissue mechanics shift between cellular- and nuclear-dominated states, driven by externally imposed deformations. (**B**) Critical strain as a function of Δε_φ_/Δε, where Δε is the excess strain and Δε_φ_ is the excess strain to the jamming transition boundary. Note that λ_c_ and λ_n_ are the cellular and the nuclear compression stiffness, and K_c_ and K_n_ are the cellular and nuclear-associated bending rigidities. Cellular-dominated mechanics show high critical buckling strain, whereas nuclear-dominated mechanics lead to lower critical strain. The crossover region facilitates the buckling instability. (**C**) Representative confocal sections of 48 hpf (top row) and 72 hpf (bottom row) retinas in Hdac1 morphants with corresponding StarDist-3D segmentation predictions. Nuclei are marked with Tg (hsp70:H2B-RFP) and cell membranes with phalloidin. Scale bar, 50 μm. (**D**) Nuclear volume distributions. See Table 3 for test statistics. (**E**) Volume fractions occupied by nuclei in ROIs and medians. Measurements in embryos treated with control morpholino (MO) at 72 hpf are split between the INL and RGL. Comparison between control MO and HDAC1 MO: at 48 hpf, n.s.; at 72 hpf (INL), *P* value < 0.01; at 72 hpf (RGL), n.s. for unpaired Student’s *t* test. (**F**) Concentration of nuclei found within the ROIs of (D) and medians. Comparison between control MO and HDAC1 MO: at 48 hpf, *P* value < 0.00001; at 72 hpf (INL), *P* value < 0.00001; at 72 hpf (RGL), *P* value < 0.01 for unpaired Student’s *t* test.

Since we showed that neuronal lamination correlates with repositioning and shape remodeling of nuclei from nematically ordered to a more crystal-like arrangement, we asked whether neuronal lamination itself could allow for more nuclei and cells to be packed within the tissue without resulting in a premature rigidity transition. To explore the effects that the nuclear packing environment can have on the mechanical state of the developing RNE, we took inspiration from a previously published continuum formulation of an epithelium growing under confinement (*58*). We followed this approach while also incorporating internal states corresponding to cellular-dominated and nuclear-dominated mechanical regimes (Fig. 5A in blue and pink, respectively). In this way, we could evaluate the separate potential contributions to the compression and bending moduli of the two-state modeled growing epithelium (see Supplementary Text). Based on this model, we observed a sudden increase in the modeled growing epithelium’s instability to buckling as it approaches the jammed, nuclear mechanics–dominated internal state, which corresponds to a sudden decrease in the critical buckling strain (Fig. 5B). Therefore, according to this model, regulation of the internal state of the RNE would directly affect tissue growth and shape and set a mechanical constraint on the proliferation of the hemispherical RNE. We thus speculated that the onset of neuronal lamination could prevent the retinal PSE from reaching a premature nuclear jamming transition, during stages in which single nuclei and cells are still actively moving through the tissue to reach their final positions (*36*, *37*, *39*, *59*). In this way, neuronal lamination would promote the transition from positional disorder to order, thereby keeping the RNE in a cellular-dominated internal state and preventing the onset of a buckling instability. This, in turn, would enable the correct growth and scaling of the optic cup.

### Blocking neurogenesis interferes with nuclear shape and size changes and leads to buckling of the retinal PSE

Based on our one-dimensional model of epithelia growing under confinement, we hypothesized that timely control of neuronal lamination ensures that the developing RNE does not approach the nuclear jamming transition, which would subsequently lead to deformations of the tissue. In this scenario, timely progression toward a laminated state could shift the threshold to entry into the nuclear-dominated mechanical state to higher nuclear packing densities and allow correct growth and shape scaling. To explore this scenario, we probed the limits of nuclear packing in the RNE by delaying neuronal lamination and thereby keeping the retina in a PSE-like arrangement for longer.

To delay neuronal lamination, we blocked neurogenesis and promoted sustained proliferation in the RNE using a previously established histone deacetylase 1 (HDAC1) morpholino knockdown (KD) approach (*21*). HDAC1 suppresses Wnt and Notch signaling in the RNE, therefore regulating the balance between proliferation and neuronal differentiation of multipotent progenitors (*60*, *61*). In hdac1^−/−^ mutants, progenitor cells do not exit the cell cycle, continue dividing, and do not differentiate into neurons, thereby blocking neuronal lamination (*60*, *61*). Consequently, hdac1^−/−^ retinal PSE shows perturbed shape and is folded at developmental stages when the control retina features a smooth surface, hemispherical shape, and delaminated tissue architecture (*60*, *61*).

We confirmed that progenitor cells in HDAC1 morphants are still organized into a PSE-like structure even at stages when the retina is already laminated in the control scenario at 72 hpf. Because progenitor cells proliferate for longer, we quantified the volume of the eye in HDAC1 morphants to follow the growth of the RNE. To do this, we used the tg (lama1:lama-GFP) transgenic line that labels the surrounding basal lamina to segment the optic cup volume and determine whether it differed between morphants and controls (fig. S7, A and B). Despite continued proliferation, our segmentation showed that the volume of the optic cup was smaller in morphants than in controls (fig. S7C). Thus, eyes in HDAC1 morphants failed to reach a normal size, likely due to failed cell elongation that allows for tissue height increase of the PSE, as previously shown (*21*). We further confirmed that, as seen before, the overall shape of the RNE starts to be visibly perturbed after 48 hpf, with the retinal PSE undergoing buckling (*21*, *60*, *61*).

We next set out to find a mechanistic explanation for the buckling phenomenon expanding on previous observational findings. To this end, we tested whether the buckling instability was influenced by changes in nuclear packing, as suggested by our theoretical model. We quantified nuclear packing densities in the RNE upon HDAC1 KD at 48 and 72 hpf, i.e., before and after buckling. Predictions obtained with our StarDist-3D model had to be manually curated and were analyzed using our image analysis pipeline (Fig. 5C). Nuclei in HDAC1 KD RNE were bigger and extremely elongated along their major axis before buckling (Fig. 5D; fig. S8, A and C; and Table 3). The extreme elongation of these nuclei suggested that they could be deformed by increasing lateral compressive stresses building up before the onset of any buckling event. Nuclear sizes were reduced only upon buckling of the PSE at 72 hpf (Fig. 5D) when nuclear aspect ratios increased by shortening of the major axis lengths (fig. S8, C and D). However, in the course of the experiment, nuclei never reached ratios seen in controls at neurogenesis onset (fig. S8D and Table 3). These results indicate that retinal progenitor cells in HDAC1 morphants could not fully adjust their nuclear size and shape as seen for progenitor cells in control embryos. Despite their extremely elongated shapes, nuclei in the HDAC1 morphants did not contact more neighbors than in the laminated retina of control embryos (fig. S8E), suggesting that the nuclei had reached the closest packing configuration for this PSE arrangement. The nuclear volume fraction in HDAC1 morphants did not increase between 48 and 72 hpf and did not reach φ_s_ as in controls, while nuclear concentrations did not increase beyond values measured at 48 hpf for control embryos (Fig. 5, E and F). This suggests that, in controls, the retinal PSE is approaching its closest packing density at around 48 hpf, when neurogenesis is ongoing and the RNE is expected to transition to a layered structure. Instead, the RNE of HDAC morphants seems to effectively approach a nuclear jamming threshold resulting in a buckling of the tissue, as suggested by our toy model (Fig. 5, A and B). Meanwhile, the retinal pigmented epithelium (RPE), which covers the apical surface of the RNE, did not buckle with the neural retina in HDAC1 morphants (fig. S8B).

**Table 3.** Volumes, axes lengths, and aspect ratios of nuclei in HDAC1 morphants. Mean and standard deviation are reported for each condition, i.e., control and HDAC1 morpholino (MO), and each developmental stage (hpf, hours post fertilization). Number of embryos (*N*) and number of nuclei in ROIs (*n*) per stage and condition: control MO at 48 hpf, *N* = 5, *n* = 12370; control MO at 72 hpf, *N* = 4, *n* = 17108; HDAC1 MO at 48 hpf, *N* = 5, *n* = 2252; HDAC1 MO at 72 hpf, *N* = 4, *n* = 4970. Mann-Whitney tests between control and HDAC1 morphants were performed both for nuclear volume and for nuclear aspect ratios distributions. *P* values are shown for each test.

Control versus Hdac1 morphants				
		Nuclear volume (μm^3^)		
Stage	MO	μ ± σ	H0: same distribution	*P* value
48 hpf	control	82.50 ± 23.12	–	–
	HDAC1	140.64 ± 45.26	Control = HDAC1	*P* ≪ 0.0001
72 hpf	control	61.56 ± 23.81	–	–
	HDAC1	99.86 ± 39.15	Control = HDAC1	*P* ≪ 0.0001
		Nuclear axis lengths (μm)		
Stage	MO	A (μ ± σ)	B (μ ± σ)	C (μ ± σ)
48 hpf	control	7.87 ± 1.29	5.03 ± 0.70	4.10 ± 0.56
	HDAC1	12.41 ± 3.35	5.19 ± 0.60	4.30 ± 0.48
72 hpf	control	6.62 ± 1.15	4.58 ± 0.77	3.87 ± 0.77
	HDAC1	9.11 ± 2.79	5.02 ± 0.74	4.22 ± 0.65
		Aspect ratio (A/ (B + C)		
Stage	MO	(μ ± σ)	H0: Same distribution	*P* value
48 hpf	control	0.59 ± 0.12	–	–
	HDAC1	0.40 ± 0.10	Control = HDAC1	*P* ≪ 0.0001
72 hpf	control	0.65 ± 0.15	–	–
	HDAC1	0.55 ± 0.15	Control = HDAC1	*P* ≪ 0.0001

To determine whether it was really the onset of neurogenesis that is important for this phenomenon, we probed whether tissue shape can be rescued when neurogenesis occurs at a later developmental stage. For this, we made use of the fact that morpholinos become progressively diluted over development, and their effects should thereby be weakened. When we injected HDAC1 morpholino in the tg (atoh7:gap-GFP) transgenic line, we were able to observe delayed neurogenesis with some atoh7-positive neuronal progenitors emerging at 72 hpf stage (fig. S7D). Nevertheless, the tissue remained in its perturbed buckled state. This shows that indeed interference with the onset of neurogenesis induces this phenotype that cannot be rescued at later developmental stages,

The phenotype of HDAC1 morphants resembles the buckling dynamics predicted by our theoretical model. This suggests that delaying neurogenesis and neuronal lamination prompts the RNE of HDAC1 morphants to transition into a nuclear-dominated mechanical state, thereby facilitating the deformation of the tissue. Our theoretical and experimental findings further indicate that tissue buckling releases some of the compressive stresses that affected nuclei in the RNE, while other tissues, such as the RPE, were not as affected. In line with this, it was shown that loss of HDAC1 activity in the hindbrain does not lead to tissue buckling (*62*), supporting our hypothesis that buckling results from deregulation of nuclear packing configurations in densely crowded PSE, such as the RNE but not in looser packed tissues. Overall, theoretical and experimental results suggest that the remodeling of nuclear shape and size during neuronal lamination is linked to the mechanical state of the tissue, therefore affecting the growth and shape of the densely crowded hemispheric PSE such as the RNE.

## DISCUSSION

In this study, we characterized nuclear packing in vivo and over development in retinal and hindbrain neuroepithelia using deep-learning methods to achieve accurate 3D instance nuclear segmentation. This enabled us to accurately detect nuclei within volumetric imaging datasets of crowded neuroepithelia and extract features that describe nuclear shape and size changes, extending previous studies that mostly performed their analyses in 2D or on small sample sizes (*15*, *32*). In this way, we generated insights into how nuclear packing in retinal PSE progressively transitions to ordered nuclear arrangements and how this can influence morphogenesis.

We find that in both retinal and hindbrain PSE the packing density of nuclei increases, but to different degrees, with the nuclear packing in the retina approaching limiting packing fractions while the hindbrain PSE staying more loosely packed. In both PSE, nuclei are arranged in a nematic-like order, indicating that this is the conserved tissue configuration of PSE. Nematic-like order means that nuclei are orientationally ordered and show strong alignment along the apicobasal axis but are positionally disordered. This organization resembles the arrangement of anisotropic particles in a nematic liquid crystal, which defines an intermediate state between a liquid phase and a solid crystal phase. The conservation of a nematic ordering of nuclei both in retinal and hindbrain PSE despite differences in proliferation, neurogenesis onset, and packing density suggests that this organization could be a beneficial feature of pseudostratification for proliferating epithelia.

PSE are commonly found as organ precursors in development but are not as widespread in the adult organism. In the context of organogenesis, one hypothesis that has been recently formulated is that smaller average cell diameters at the apical surface could enhance the precision of morphogen-based patterning and that pseudostratification itself could be advantageous because it promotes the reduction of cellular cross-sectional areas (*25*, *63*). Complementary to this hypothesis, we propose that the nematic ordering of nuclei could facilitate the radial nuclear translocations that characterize proliferating PSE, regardless of high nuclear packing densities. Apical nuclear movements have been shown to come with extensive cell rearrangements, therefore maintaining tissue fluidity in the proliferating mouse neural tube neuroepithelium (*26*). Therefore, the nematic-like order could represent one of the advantages that characterize pseudostratification and that facilitate nuclear movements along the apicobasal cell axis to keep the fluidity of the tissue. Moreover, we propose that this, together with the elongated cell shapes, could allow for dense packing of neuroepithelial cells in proliferating PSE without reaching a premature nuclear jamming transition as suggested in different words by I. Smart about 50 years ago (*1*).

A nuclear jamming transition has been recently reported in 2D simulations of epithelia during development (*32*). Here, cells were shown to become progressively constrained when the nuclear volume fraction increased and nuclear anisotropy decreased. In that study, comparison of the aforementioned in silico findings to 2D optical slices of the zebrafish retina suggested that nuclei in the INL reached a jamming transition boundary between 55 and 72 hpf, similarly to our observations. Additional to the interpretations of this 2D analysis, that we experimentally confirmed, our 3D analysis of nuclear packing allowed us to decipher how nuclear arrangements in the RNE transition from nematic order to a fully crystal-like periodic order while still retaining long-range alignment. We showed that the observed changes in nuclear shape anisotropy occur after neuronal differentiation and that timely onset of neuronal lamination enables correct tissue growth and morphology. At the same time, neuronal lamination avoids the onset of a compressive buckling instability that would be strongly enhanced by the approach to a nuclear-dominated mechanical state. These findings should be further compared to other systems in which the role of nuclear shape and positioning can affect the remodeling of tissue topologies of other developing epithelia, such as in the germband of *Drosophila* embryos (*64*).

Over the course of neuronal lamination in the retina, nuclei of neuronal precursors change their shape, size, and position, leading to the emergence of crystal-like arrangements. We find that most nuclei across the three nuclear layers, with the exception of horizontal cells, are still aligned along the radial axis of the tissue. This coincides with the physiological direction of light propagation. Because photons need to pass through the whole thickness of the retina to reach the photoreceptors, correct and precise vision in vertebrates is aided by minimal light scattering. It has been shown that scattering is reduced by the light-guiding capabilities of the Müller glia (*65*, *66*) and by precise spatial organization of heterochromatin of rod photoreceptors in nocturnal animals (*67*, *68*). Our study indicates that nuclei in the retina are arranged with orientational and crystalline order, which could further enhance tissue transparency and light propagation, as previously suggested by simulations of ordered nuclear arrangements (*67*). Future studies should explore how these arrangements differ in the brain, which originates from pseudostratified neuroepithelia but is generally more opaque then the retina.

The presented 3D analysis allowed us to characterize nuclear shape and size changes over the course of retinal development and explore how these changes, together with positioning and alignment, could determine nuclear packing densities. When we probed the limits of the retinal PSE upon perturbation of neurogenesis, we found that nuclear packing in the RNE could not reach higher volume fractions without transitioning to a layered tissue. This suggests that in the vertebrate retina, the limiting nuclear packing for a PSE is reached around the time of neuronal lamination. When blocking neurogenesis and neuronal lamination by the HDAC1 KD, severe tissue deformations were observed. This resembles the phenotype described by several theoretical and experimental studies proposing that proliferation of epithelia under confinement can induce buckling instabilities, i.e., without the requirement of active bending mechanisms (*58*, *69*, *70*). Epithelial cells can partially accommodate compressive stresses by increasing their height until the critical strain for the compressive instability is reached, after which the tissue buckles (*69*). Progenitor cells in HDAC1 morphants are characterized by extremely elongated nuclei before buckling of the RNE, while they fail to increase their cell height (*21*). Meanwhile, the RPE is stretched over the RNE, except for in those areas where the retina has buckled. We speculate that the RPE could confine the growth of the retina and therefore contribute to the buckling phenotype from the outside. Consequently, we propose that the strong nuclear anisotropy we observe upon HDAC1 KD is due to the compressive stresses generated by the proliferating cells under confinement, until these stresses cannot be accommodated further for dense nuclear packing in a PSE-like arrangement, leading to tissue buckling. This is in line with the predictions from our 1D continuum model that shows that the approach to a nuclear mechanics–dominated state further facilitates tissue buckling. Based on this, we propose that neuronal lamination circumvents the buckling instability in the retina by keeping the tissue sufficiently in a looser, cellular mechanics–dominated state.

In conclusion, our study shows that pseudostratification establishes a nematic-like order early on during organogenesis and that the emergence of crystal-like nuclear arrangements enables close nuclear packing of newly formed neurons without affecting tissue shape. These insights were only possible due to the 3D nuclear segmentation over the entire proliferative and neurogenic phases. Because zebrafish is characterized by a particularly fast-paced embryogenesis, it will be interesting to compare these findings in the zebrafish RNE to other teleosts and vertebrates that develop over longer timescales. For instance, future studies could dissect the distribution of forces and stresses within crowded or not-so-crowded neuroepithelia across different species and compare how timely the regulation of proliferation and neurogenesis can lead to different tissue shapes as a result of buckling occurring either in a nuclear-dominated or cellular-dominated regime. This will inform us about the mechanisms that control nuclear packing order in other neurodevelopmental contexts and in other tissues and to what extent mechanical feedbacks and instabilities linked to the nuclear packing states are important for guiding and determining tissue shape.

## MATERIALS AND METHODS

### Zebrafish husbandry

The experimental work reported in this article was performed on *Danio rerio*, i.e., zebrafish, embryos aged between 24 and 80 hpf. WT [AB and Tupfel long-fin (TL) strains] and transgenic fish were bred and maintained in a recirculation life support system (Tecniplast) with the following parameters: 28°C, pH 7.0, conductivity of 1000 μS/cm, and 14-hour light/10-hour dark cycle. Up to juvenile stage, fish were fed with a combination of saltwater rotifers (*Brachionus plicatilis*) and processed diet (Gemma 150, Skretting). Adult fish were fed with a combination of live food (*Artemia salina*) and commercial processed dry food (Gemma 300, Skretting). Embryos used for experimental work were raised at 21°, 28.5°, or 32°C in E3 medium supplemented with 0.2 mM 1-phenyl-2-thiourea (10107703, Acros Organics) from 8 hpf to prevent pigmentation. Embryos were staged according to Kimmel *et al.* (*71*). Anesthesia was performed by supplementing the E3 medium with 0.04% tricaine methanesulfonate (MS-222, 1004671, Pharmaq) before live imaging. All animal work was conducted in accordance with institutional standard operating procedures under the licensing of the DGAV (Direcção Geral de Alimentação e Veterinária, Portugal) and in accordance with the European Union (EU) directive 2010/63/EU and with the Portuguese Decree Law no. 113/2013.

### Transgenic lines

To visualize all nuclei in the retina, the Tg (β-act:H2A-GFP) (*72*) and the Tg (hsp70:H2B-RFP) (*73*) line were used. Tg (atoh7:gap-GFP) zebrafish transgenic line was used to identify Atoh7^+^ progenitors and Atoh7^+^ neurons (*74*). The Tg (lama1:lama1-sfGFP) was used to visualize the outline of the optic cup (*75*).

### Heat shock of embryos

Transgenic lines expressing constructs under the heat shock promoter (hsp70) were incubated in water bath set to 37.5°C for at least 20 min to drive the expression of the construct. In the case of Tg (hsp70::H2B-RFP), embryos were heat-shocked for 20 to 30 min, and imaging was started around 3 to 4 hours after. For embryos analyzed at stages beyond 36 hpf, the heat shock was performed twice to enhance the signal: one time the day before the experiment and a second time 4 hours before imaging or fixing.

### Morpholino experiments

All morpholinos were purchased from Gene Tools LLC. Morpholino targeting HDAC1 (5′-TTGTTCCTTGAGAACTCAGCGCCAT-3′) was injected at 0.5 ng per embryo, together with the morpholino targeting p53 (5′-GCGCCATTGCTTTGCAAGAATTG-3′), which was added to the injection mix (0.75 ng per embryo) to alleviate cell death possibly resulting from the off-target effects of the morpholino. Control embryos were injected with a scrambled morpholino (5′-CCTCTTACCTCAGTTACAATTTATA-3′) injected at 0.5 ng per embryo, together with the p53 morpholino.

### Whole mount immunofluorescence

All immunostainings were performed on whole-mount embryos fixed overnight in 4% paraformaldehyde (043368-9 M, Thermo Fisher Scientific) in 1× PBS at 4°C as previously described (*35*, *36*). Embryos were washed from three to five times for 10 min in 0.8% PBS-Triton (28817295, VWR) and permeabilized in 0.25% Trypsin-EDTA (sc-391060, Santa Cruz Biotechnology) on ice for different time periods depending on the developmental stage: 10 min for 24, 28, and 36 hpf; 12 min for 42 hpf; 15 min for 48, 60, 72, and 120 hpf. Embryos were then washed for 30 min on ice in 0.8% PBS-Triton on shaker and then incubated in blocking solution, i.e., 10% goat serum (16210064, Gibco) in 0.8% PBS-Triton, for 3 hours at room temperature. Embryos were then incubated with the following primary antibodies for 72 hours at 4°C on shaker: anti-GFP (1:100; Proteintech, 50430-2-AP), histone H3 phosphorylated on S28 (1:500; Abcam, ab10543), anti-HuC/HuD (1:250; Invitrogen, A-21271). Embryos were washed three times for 30 min with 0.8% PBS-Triton and then incubated for 24 to 48 hours with fluorescently labeled secondary antibodies (Molecular Probes). In the case of the Tg (hsp70::H2B-RFP) transgenic line, embryos were incubated in antibody solution with RFP booster (1:200; ChromoTek, rba594) to enhance the fluorescence signal of the expressed RFP. Phalloidin conjugated with Alexa Fluorophore 405 (1:100; Biotium; 00034-T), Alexa Fluorophore 488 (1:50; Life Technologies; A12379), and Alexa Fluorophore 647 (1:50; Cell Signaling Tehcnology, 8940) were used to label F-actin in all cells. Last, embryos were washed several times with 0.8% PBS-Triton in ice on shaker and stored in PBS at 4°C until imaging.

### Laser scanning confocal microscopy

Dechorionated, fixed and immunostained samples were imaged with a Zeiss LSM980 Airyscan2 inverted microscope, equipped with two PMT and one GaAsP detector using a 40×/1.1 C-Apochromat water immersion objective from Zeiss. Embryos were mounted in 0.70% low-melting agarose in glass-bottomed dishes (35 mm; MatTek Corporation) and imaged at room temperature. The dataset used for nuclear segmentation was acquired with Airyscan CO-8Y mode and processed using the Airyscan processing methods for 3D datasets. The microscope was operated using the proprietary ZEN Blue v3.3 software.

### Light sheet fluorescence microscopy

Dechorionated live tg (lama1:lama1-sfGFP) embryos were mounted in 1-mm glass capillaries in 0.6% low-melting agarose. The sample chamber was filled with E3 medium containing 0.01% MS-222 (Sigma-Aldrich) and 0.2 mM propylthiouracil (10107703, Acros Organics). Imaging was performed on a Zeiss Lightsheet Z.1 microscope equipped with two PCO Edge 4.2 scientific complementary metal-oxide semiconductor cameras (max, 30 fps with 2048 × 2048 pixels) and with a 20×/1.2 Zeiss Plan-Apochromat water-immersion objective. Z-stacks spanning the entire RNE were acquired with 1-μm optical sectioning with double-sided illumination mode. The microscope was operating using the proprietary ZEN Black v3.0 software.

### Segmentation of the optic cup

The 3D segmentation of the basement membrane was performed using the Fiji (*76*) plugin for 3D segmentation LimeSeg (*77*) (v 0.4.2). The outline of the optic cup was marked by the tg (lama1:lama1-sfGFP) line. Several regions of interest (ROIs) where drawn inside the volume of the optic cup across the z-stack. End points were defined by a single point. All ROIs were saved in the Fiji ROI manager and sorted according to their position along the *z* axis. The “Skeleton Seg” approach in LimeSeg was used with the following parameters: D0 (minimal diameter of smallest object in pixel) equal to 16, *F* pressure (default pressure) of 0.0099, range in d0 units equal to 1, and number of integration steps of −1. The resulting 3D mesh of the object’s surface was saved in ply format, and a python script was coded to convert this mesh into a labeled mask in tiff format (the script can be found in the github repository of this study). The resulting labeled objects were then manually corrected using the label tools in Napari (*78*) (v0.4.18).

### 3D instance segmentation of nuclei

Deep-learning methods generally require vast amounts of pixel-wise annotated ground truth data for training. For this reason, we manually annotated up to 16 ground-truth 3D cropped images of various sizes, at least 50 × 260 × 230 pixels having voxels size equal to *zyx* = [0.24, 0.102, 0.102] in micrometer, taken from several datasets representing various developmental stages. The stacks sizes were defined by the volume of the nuclei, since each crop needed to be big enough to contain fully visible nuclei, i.e., nuclei not touching the image border. Manual annotation was performed using the label tools in Napari.

Using the aforementioned annotated stacks, we trained two StarDist-3D models: One model (model A) was trained on 16 ground-truth stacks representing stages between 24 and 72 hpf, while the other model (model B) was trained on 14 stacks representing later stages of the laminated retina, i.e., between 48 and 80 hpf. Each stack contained between 10 and up to 110 entire labeled objects, i.e., nuclei that were not touching the image border. In both training datasets, the *z* step was twice the voxel size in *xy*, and this anisotropic factor was kept in the imaging dataset. The voxel size used was comparable to the voxels size in the training datasets. To artificially increase the size of the training dataset and make the model more robust to pixel intensity fluctuations, the training dataset was augmented by adding synthetical noise on all pixels and rotating, flipping, and transforming each labeled image. The following parameters were used to optimize the neural network for the training: number of rays, 256; grid size, [1, 4, 4]; anisotropy, [4.17, 1.27, 1.0]; backbone, u-net; u-net pool, [2, 4, 4]; train patch size, [32, 128, 128]; epoch; 400; steps per epoch, 100; and train learning rate, 0.003. The script coded to perform the StarDist-3D training on our data can be found in the github repository of this study and can be reused under open-source license conditions.

To evaluate the trained models, predictions were run on another manually annotated dataset that had never been shown to StarDist-3D during training. Table S1 shows the accuracy, precision, and recall for several intersection over union thresholds τ for test datasets representing distinct developmental stages, i.e., 30, 48, and 72 hpf. The number of test ground-truth crops per stage was, respectively, *N* = 2, *N* = 3, and *N* = 4. The total number of nuclei per stage, respectively, is *n* = 272, *n* = 225, and *n* = 647. StarDist-3D model A was tested on datasets from 30 and 48 hpf embryos, while model B was tested on dataset from 72 hpf embryos (table S1).

Since StarDist-3D is well suited for objects that can be represented by star-convex polyhedra, such as in the case of round and elliptical nuclei, our models could not produce accurate predictions of extremely elongated nuclei. This was the case for some of the elongated nuclei in WT retinas at 24 hpf and in HDAC1 morphants. Therefore, the imaging dataset of retinas from these samples was segmented using our StarDist-3D models, and the predictions were manually curated using Napari (v0.4.18).

### Feature extraction

StarDist-3D provided as an output 32-bit float tif files that were converted to integer data type. Each segmented nucleus was identified by an integer value, and all segmented nuclei touching the borders of the image were excluded from the analysis. The images were rescaled to obtain almost isovolumetric voxels that would facilitate the extraction of some of the features of interests. The script iterated through all nuclei in the image to measure the volume; the longer, intermediate, and shorter axes from the centroid; the primary and secondary nematic axes; and the centroid coordinates. The primary nematic was calculated as the longest pairwise distance between points composing the nuclear boundaries. The secondary nematic was defined as the maximum pairwise distance between points of the nuclear boundaries lying on the midplane to the primary nematic. Because nuclei supposedly do not touch each other, the nuclear boundaries of each nucleus were dilated to count the number of touching neighbors at different radii. To compute the neighbor statistics of each nucleus, a touch matrix was generated, reporting the centroid-to-centroid distance for each touching neighbor. In this way, the touching and proximal neighbors and the internuclear distances were computed for each nucleus in the ROI. The pipeline was coded in Python 3.9 and mainly based on the following packages: numpy, skimage, and pyclesperanto (*79*–*82*).

### Image smoothing of extracted features

To explore the local spatial variations of extracted features, an automatic script was written to scan each image StarDist-3D prediction with a 3D window of a given size (fig. S2B). The images shown in the main figures were obtained using a 10 μm–by–10 μm–by–10 μm size window. The mean value for the feature of choice was measured within the volumetric window, e.g., the average number of contacts. Because the volumetric windows were overlapping by 20%, each pixel was averaged by the times the window looped on it. This created a smoothed image stack where each pixel reported the mean value for the measurement of interest (fig. S2C). This analysis was coded in Python 3.9 and mainly based on the following packages: numpy, skimage, and pyclesperanto (*79*–*82*).

### Selection of the region of interests

The ROIs were selected in the central part of the retina. This allowed to segment nuclei belonging to cells that were at similar developmental stages and differentiation trajectory. To perform this across different retinae, the lens was used as a reference for depth (movie S3), and the ROIs were manually selected using a Python script that enables the visualization and user interaction with the dataset using Napari (the script can be found in the github repository of this study). In this way, the user could draw a polygon over the area of interest and specify the range of slices desired. The script iterated through all the labeled nuclei present within this region and created a new image stack containing only those nuclei. Then, another Napari graphical user interface (GUI) automatically opened to allow the user to manually mark the labeled nuclei that were erroneously included in the new image. Ultimately, a csv file reporting the labels of nuclei contained in the selected ROI was produced.

### Analysis of the orientational order of nuclei

The description of a nematic liquid crystals involves the quantification of the order in the system. The average angle between the primary nematic axis of each nucleus and the primary nematics of its touching neighbors was calculated. The calculated averaged angles were used to compute the scalar order parameter S in selected ROIs

S=(3/2)⟨cos2 θ⟩−1/2

where θ is the angle between the liquid-crystal molecular axis and the local director. Note that for a completely random and isotropic sample, *S* = 0, whereas for a perfectly aligned sample, *S* = 1. For a typical nematic liquid crystal sample, 0.4 < *S* < 0.9. The same procedure was performed for the secondary nematic axes.

### Analysis of the RDF

The RDF 𝑔 (𝑟) provides a statistical description of the local packing and particle density of a system. It is defined as

$$g(r)=n(r)/n0\;4\pi r^{2}\;dr$$

where 𝑛 (𝑟) is the number of particles in a spherical shell of radius 𝑟 and thickness 𝑑𝑟, 𝑛0 is the average number density of particles, and 4π𝑟2 𝑑𝑟 is the volume of the spherical shell. The 𝑔 (𝑟) was calculated iteratively for the increment 𝑑𝑟 steadily increasing the radius of the external spherical shell, with a maximal radius of 50 μm. To calculate the number density of nuclei within the ROI, the nuclear boundaries were dilated so that the space between the nuclei would be completely filled. Then, this labeled object was eroded to mask the nuclei in the ROI. The number density was calculated as the ratio between the number of nuclei in the mask and the volume of the mask itself. No compensation for edge effects was performed.

### Statistical analysis

All statistical tests used are indicated in the figure or table legends and the definitions of error bars. Likewise, *P* values and sample sizes are reported in the figure and table legends or in dedicated tables. After visual inspection of nuclei in the selected ROIs, a lower and upper threshold of nuclear volumes was used to filter the data obtained after extraction of all features of interest from the StarDist-3D predictions. More specifically, we used a lower threshold of 50 μm^3^ and upper thresholds of 400 μm^3^ for nuclei in embryos staged between 24 and 48 hpf. This was done to reduce the number of erroneously segmented nuclei. Data visualization and statistical analysis were performed using the Matplotlib, seaborn, and scipy statistics packages in Python 3. Further information about the exact libraries, together with their versions, are detailed in the Github repository.

## Supplementary Materials

### The PDF file includes:

Supplementary Text Figs. S1 to S8 Table S1 Legends for movies S1 to S3 References
