# Supplementary material for: Timely neurogenesis drives the transition from nematic to crystalline nuclear packing during retinal morphogenesis
Source: Sci Adv. 2025 May 9;11(19):eadu6843. doi: 10.1126/sciadv.adu6843 (PMC12063663; doi:10.1126/sciadv.adu6843)
Supplement: Supplementary file 1 — Supplementary Text Figs. S1 to S8 Table S1 Legends for movies S1 to S3 References [file sciadv.adu6843_sm.pdf]

Science Advances
AAAS

Supplementary Materials for

Timely neurogenesis drives the transition from nematic to crystalline nuclear packing during retinal morphogenesis

Lucrezia C. Ferme *et al.*

Corresponding author: Lucrezia C. Ferme, lucrezia.ferme@gimm.pt; Caren Norden, caren.norden@gimm.pt

Sci. Adv. **11**, eadu6843 (2025)
DOI: 10.1126/sciadv.adu6843

The PDF file includes:

Supplementary Text
Figs. S1 to S8
Table S1
Legends for movies S1 to S3
References

Other Supplementary Material for this manuscript includes the following:

Movies S1 to S3

Nuclear Packing Buckling Instability Theory Supplement

1 Introduction and Set-Up

In order to model the effect that the nuclear packing environment in the optic cup has on tissue stability and the potential for a buckling transition, we take inspiration from Trushko et al. (2020) [57]. There, Trushko and coauthors ren-der a leading-order continuum model of an epithelium growing under spherical confinement as an (initially) circular elastic ring confined in a circular geometry of radius R. When the elastic ring becomes larger than the size of the con-fining circle, a buckling instability sets in and the elastic ring breaks circular symmetry and buckles. More in depth treatments of this problem have also been carried out to higher order, for example by Napoli and Turzi [83], although the dominant, leading order behavior is unchanged. Here, we choose to follow the simpler approach of Trushko et al., but additionally capture the nuclear packing environment in the coarse-grained continuum model as two distinct material states, with material properties dependent in a switch-like manner on the packing fraction.

We begin by adapting the global energy per unit length of the confined 1d elastica ring, for L the current length of the elastica and L_0 the equilibrium (preferred) length without any constraints:

$$E = K \int_L \kappa^2 ds + \lambda \int_{L_0} \left(\left| \frac{\partial \mathbf{r}}{\partial s_0} \right| - 1 \right)^2 ds_0 \tag{1}$$

where we chose here to ignore any potential energetic contribution from dis-tortions to the confining medium and assume instead that the combination of ECM and other tissues around the optic cup are significantly stiffer and ef-fectively incompressible. The first term captures the bending energy of the elastica, while the second term captures any contribution from compression (or extension) along the tissue.

In order to derive a critical strain for the onset of the buckling instability and investigate how a two-state system of internal stiffnesses in the tissue affects this instability, we will need a simple, minimal buckled elastica shape (confined within a circle) to compare with the elastica remaining circular at the cost of

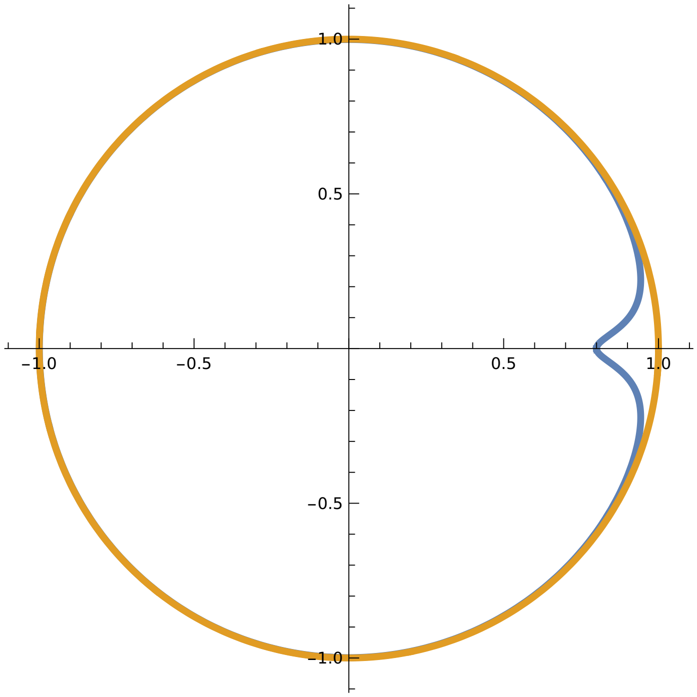

Fig. TS1: Example buckled configuration, shown here for an elastica (blue) constrained inside a rigid unit circle (orange), with $\delta = 0.2$ and $\alpha = 0.05$.

compression energy. As Trushko et al. do, we also choose to use a simple trigonometric representation of the initially buckled shape:

$$r(\theta) = R\left(1 - \frac{\delta}{1 + (\sin(\theta/2)/\alpha)^2}\right). \tag{2}$$

Unlike Trushko et al., though, we assume that the buckle occurs at $\theta = 0$ and that there is only one buckling site, requiring the extra factor of 1/2 inside the argument of the sine function. Here, α and δ are dimensionless parameters controlling the shape of the buckled region, with δ setting the depth of the buckled fold and α setting the width of the buckled region. The length of an elastica parameterized in this way is given by:

$$L_0 = \int_0^{2\pi} \sqrt{(x(\theta)')^2 + (y(\theta)')^2}d\theta. \tag{3}$$

As we intend to compare a just-buckled elastica shape with its compressed, pre-buckled counterpart, we may assume that the amplitude and extent of the buckled region are small and we accordingly set $\alpha, \delta \ll 1$. We may then expand in small quantities, allowing us the write, to leading order:

$$L_0 = 2\pi R\left(1 + \frac{\delta^2}{32\alpha} - \delta\alpha\right). \quad (4)$$

It is useful here to introduce the excess strain, $\Delta\epsilon = (L_0 - 2\pi R)/2\pi R$, leading to:

$$\Delta\epsilon = \frac{\delta^2}{32\alpha} - \delta\alpha. \quad (5)$$

Continuing to follow Trushko et al., we treat the expression for the bending energy similarly, expanding to leading order in small quantities, and writing:

$$E_b = \frac{2\pi K}{R}\left(1 + \frac{3\delta^2}{64\alpha^3}\right). \quad (6)$$

Note that naively taking our small quantities back to zero here is suddenly problematic as it would imply a diverging energy! This is an early hint that the scaling of δ and α are not independent of one another for a physically realistic buckled region, and that δ should scale faster than α in order to cut off the unphysical divergence. How to ensure that we are considering a physically realistic buckled region, then? We wish to work with the elastica shapes that, for a given excess strain $\Delta\epsilon$, minimize the bending energy. We can write the Lagrangian for this optimization as:

$$\mathcal{L} = \frac{2\pi K}{R}\left(1 + \frac{3\delta^2}{64\alpha^3}\right) + \ell\left(\frac{\delta^2}{32\alpha} - \delta\alpha - \Delta\epsilon\right) \quad (7)$$

with ℓ the Lagrange multiplier enforcing the condition arising from the given excess strain. Computing the requisite gradients and solving for δ and α in terms of $\Delta\epsilon$ finally yields the following scaling relations:

$$\delta = 4(10^{1/3})\Delta\epsilon^{2/3} \quad (8)$$

$$\alpha = (10^{-1/3})\Delta\epsilon^{1/3} \quad (9)$$

which may be then re-substituted back into the expression for the bending energy to obtain the scaling of the pure-bend configuration's energy with $\Delta\epsilon$:

$$\frac{E_b - 2\pi K/R}{2\pi K/R} = \frac{15}{2}(10^{2/3})\Delta\epsilon^{1/3} \quad (10)$$

Meanwhile, the elastic compressive energy of the confined elastica is simply quadratic in the excess strain, as it would be for a spring or any other Hookean material, and the pure-compression configuration's energy scales with $\Delta\epsilon$ as:

$$E_s - 2\pi K/R = \lambda(2\pi R)\Delta\epsilon^2. \quad (11)$$

Initially, for very small values of $\Delta\epsilon$, the pure-compression configuration is less energetically costly and, despite the fact that there is some excess strain, the elastica does not buckle to adopt curvature. At larger values of $\Delta\epsilon$, however, the

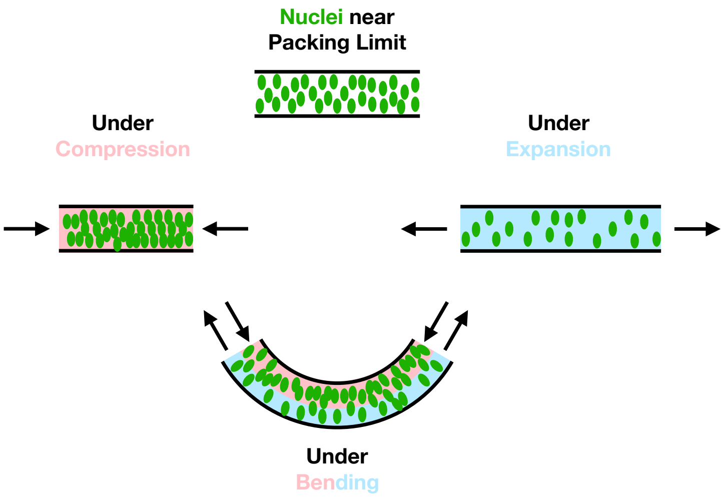

Fig. TS2: Schematic indicating the interplay of the internal packing state of the tissue's nuclei with externally imposed deformations: compression, expansion, and bending. Compression can drive the mechanics from cellularly dominated to nuclearly dominated while expansion is the reverse. Bending creates a mix of both.

pure-bend configuration is favored over pure compression and buckling would therefore be expected to take place. Since the energetic costs of the two possible configurations switch places in relative costliness as $\Delta\epsilon$ increases, there must be a critical value of the excess strain, $\Delta\epsilon_c$, where the two energies are equal. This $\Delta\epsilon_c$ therefore captures the onset of the buckling instability and can be found by setting $E_b = E_s$:

$$\Delta\epsilon_c^{5/3} = \frac{15}{2}(10^{2/3})\frac{K}{\lambda R^2}. \tag{12}$$

Note that for a fixed size of the constraining environment, the critical excess strain required to initiate buckling is thus controlled entirely by $(K/\lambda)^{3/5}$. We have now gathered the necessary ingredients to consider the two-state-stiffness tissue we propose as an analogy for the developing RNE.

2 Nuclear jamming and the onset of buckling

To this point we have closely followed the approach of Trushko et al. in deriving the scaling of the energies and the critical buckling excess strain for the constrained elastica. In order to incorporate multiple internal states into the elastica that depend on $\Delta\epsilon$ and may alter its stiffness, however, now requires us to set off in a new direction. We wish to define new compression and bending moduli, λ and K that are functions of the preferred length, L_0, of the elastica in a way that captures the onset of nuclear jamming in the RNE and the attendant switch from *cellular*-associated stiffness in the unjammed state to *nuclear*-associated stiffness in the jammed state. We write:

$$\lambda(L_0) = \lambda_C + (\lambda_N - \lambda_C)H(L_\phi - L_0) \tag{13}$$

for λ_C the cellular compression stiffness, λ_N the nuclear compression stiffness, L_ϕ the tissue length below which jamming occurs given the number of nuclei present, and $H()$ the Heaviside step function. We further assume that $\lambda_N > \lambda_C$, as the nuclear envelope is commonly understood to be the stiffest component of the cell, often in the range of 2-10 times stiffer than the rest of the cell, and in many cases one might even expect $\lambda_N \gg \lambda_C$ [29, 30]. We also introduce an L_0-dependence to the bending rigidity in a similar manner:

$$K(L_0) = K_C + (K_N - K_C)H(L_\phi - L_0) \tag{14}$$

for K_C and K_N the cellular and nuclear-associated bending rigidities, respectively. Recall that the bending rigidity generically scales with the compression/stretching stiffness (i.e. the Young's modulus), $K \sim \lambda$, for simple homogeneous isotropic materials. This is because one can think of the source of bending energy for a downward directed bend as a profile of stretch above the mid-line (or mid-plane for a bending plate or cantilever) and compression below. In the regime of our tissue dominated by the cellular mechanics, where the nuclei are not densely packed enough to begin mechanically interacting, this same simple scaling holds and we may write $K_C \sim \lambda_C$. However, something more interesting happens in the close-packed, nuclear-jammed state. Here, under a bending deformation, the tissue on the side of the mid-line experiencing compression remains in the regime controlled by the nuclear mechanics, but on the opposite side, where the tissue is stretched and expanded, the nuclei fall out of their jammed state and cellular mechanics dominate. The scaling of K_N is therefore more subtle, and can be realized as $K_N \sim (\lambda_C + \lambda_N)/2$ so long as the system is not too deep into the jammed regime even at relaxed length, since exactly half the tissue experiences one compression/stretching stiffness and the other half experiences the other. For convenience we employ a scale factor, $k = h^3/12(1-\nu^2)$, for h the tissue height and ν its Poisson ratio in order to capture the rest of the bending rigidity [84] and write:

$$K_C = \lambda_C k\,;\ K_N = \frac{\lambda_C + \lambda_N}{2} k \tag{15}$$

Let us now consider the different possible relevant regimes of $L_0, 2\pi R$, and L_ϕ. We may begin by enforcing the assumption that $L_0 > 2\pi R$ as otherwise there is no longer any effective confinement and nothing interesting happens. Thus there are three remaining regimes to investigate: (1) $L_\phi < 2\pi R < L_0$, (2) $2\pi R < L_\phi < L_0$, and (3) $2\pi R < L_0 < L_\phi$.

In the first regime, all the other length scales in the system are greater than the length below which jamming and the attendant nuclear-associated stiffnesses set in and the tissue is solidly in the cellular mechanics dominated state. The critical buckling excess strain is therefore given by:

$$\text{If } L_\phi < 2\pi R < L_0: \ \Delta\epsilon_c^{5/3} = \frac{15}{2}(10^{2/3})\frac{k}{R^2}. \tag{16}$$

In the third regime, on the other hand, the other length scales in the system are below the jamming threshold length, L_ϕ and thus the tissue is solidly in the nuclear mechanics dominated state. Now, the critical buckling excess strain is instead given by:

$$\text{If } 2\pi R < L_0 < L_\phi: \ \Delta\epsilon_c^{5/3} = \frac{15}{2}(10^{2/3})\left(\frac{\lambda_C + \lambda_N}{2\lambda_N}\right)\frac{k}{R^2}. \tag{17}$$

Note that the critical excess strain has decreased here relative to its value in the first regime, perhaps significantly so if $\lambda_N \gg \lambda_C$, meaning that buckling becomes easier to initiate once the tissue is (nuclearly) jammed.

What of the second, intermediate regime? Here the pure-bend configuration has tissue length $L_0 > L_\phi$ and resides in the cellular mechanics dominated state, while the pure-compression configuration has tissue length $2\pi R < L_\phi$ and resides in the nuclear mechanics dominated state. However, the compressional energy we used earlier to derive $\Delta\sigma_c$ must be adjusted in this case, as compressing the tissue from its preferred length L_0 down to the current, unbuckled length $2\pi R$ requires crossing the L_ϕ boundary and switching stiffness regimes. In other words, some of the work done compressing the tissue is done in the cellular mechanics dominated state, even if the tissue is currently jammed in the nuclear mechanics dominated state. The corrected stretch/compression energy now reads:

$$E_s - 2\pi K/R = \frac{1}{2}\lambda_C(L_0 - L_\phi)^2 + \lambda_C(L_0 - L_\phi)(L_\phi - 2\pi R) + \frac{1}{2}\lambda_N(L_\phi - 2\pi R)^2 \tag{18}$$

or, rearranging and re-writing the per-unit-length energy in terms of $\Delta\epsilon$ gives:

$$\mathcal{E}_s = \frac{1}{2}\lambda_C(2\pi R)\Delta\epsilon^2 + \frac{1}{2}(\lambda_N - \lambda_C)(2\pi R)\Delta\epsilon_\phi^2 \tag{19}$$

where $\Delta\epsilon_\phi$ is the excess strain to the jamming transition boundary, $\Delta\epsilon_\phi = (L_\phi - 2\pi R)/2\pi R$. We can now use this new energy to derive the adjusted critical excess strain in this crossover regime, finding:

$$\text{If } 2\pi R < L_\phi < L_0: \ \Delta\epsilon_c^{5/3} = \frac{15}{2}(10^{2/3})\frac{\lambda_C}{g^2\lambda_N + (1-g^2)\lambda_C}\frac{k}{R^2} \tag{20}$$

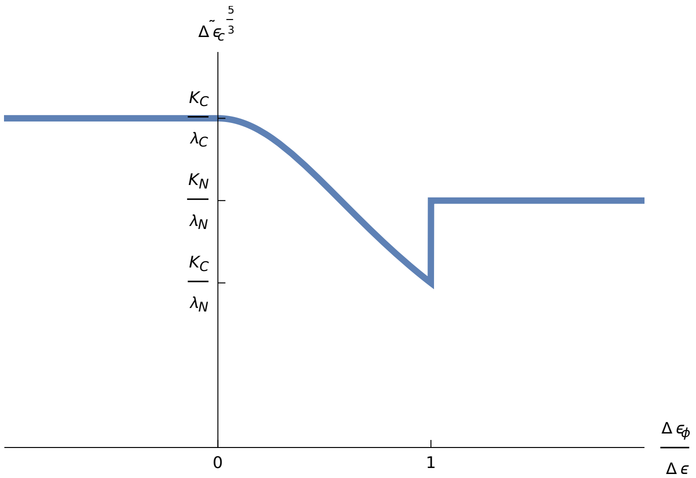

Fig. TS3: The normalized critical strain, $\Delta\tilde{\epsilon}^{3/5} = R^2\Delta\epsilon_c^{3/5}(2/15)10^{-2/3}$ as a function of g. Note that the critical strain is relatively high in the regime dominated by cellular mechanics and relatively low in the regime dominated by nuclear mechanics. In the crossover region, on approach to the nuclear-mechanics dominated regime, the onset of a buckling instability becomes even easier still, with a decrease of $\Delta\tilde{\epsilon}^{3/5}$ by a factor of 10, for examplee, if $\lambda_N \sim 10\lambda_C$, which is in the physiologically relevant range [3, 4].

for $g = \Delta\epsilon_\phi/\Delta\epsilon$. Note that $0 < g < 1$, as $\Delta\epsilon_\phi$ ranges from 0 to $\Delta\epsilon$ as L_ϕ ranges from $2\pi R$ to L_0. Note further that, in this regime, as $g \to 1$, $\Delta\epsilon_c^{5/3} \to \lambda_C/\lambda_N$ and not K_C/λ_N, leaving a discontinuous jump in the behavior of the critical exponent, as can be seen in Fig. TS3. A more complex, quasi-2D shell treatment of the problem that includes the varying amounts of extension and compression due to bend away from the mid-line of the elastica for different thicknesses would be expected to resolve this discontinuity by introducing a term coupling the stretch and the bend, but would also be expected to retain the same qualitative feature of the critical exponent diving down below the value for nuclear-mechanics dominated regime then sharply recovering to match that value very close to $\Delta\epsilon_\phi = \Delta\epsilon$. Such a shell theoretic treatment of a system with strain-dependent two-state material properties is beyond the scope of what is required to understand the optic cup buckling susceptibility here, but nevertheless constitutes an interesting direction for future theoretical work.

Returning to the case of the developing hemispheric pseudostratified retinal neuroepithelium, the fact that the approach to a jammed, nuclear-mechanics dominated internal state is accompanied by a sharply decreased critical buckling strain presents a harsh mechanical constraint on the proliferation and arrangement of the RNE as the optic cup develops. Indeed, if the developmental program wishes to ensure that the shape of the optic cup is preserved – as it must be in order to guarantee proper function of the eye – then the final approach to

the nuclear-mechanics dominated internal state is to be avoided. Strikingly, in the wildtype optic cup, it is precisely when the nuclear packings begin to approach limiting values that neurogenesis and lamination begin, and the nuclear arrangements are allowed to adopt spatial order in addition to their pre-existing orientational order. These more crystalline packing states are associated with significantly higher free volumes than in the disordered cases, opening up more room for the nuclei at the same densities, and allowing the tissue to remain in the cellular mechanics dominated regime, relatively safe from the onset of buckling instabilities. This analysis therefore predicts that, if the transition to neurogenesis and lamination is interfered with, then the growing optic cup may no longer be able to avoid the marked decrease in the critical buckling strain, and uncontrolled, ectopic buckles and folds would very likely result, matching what we observe in the HDAC mutants.

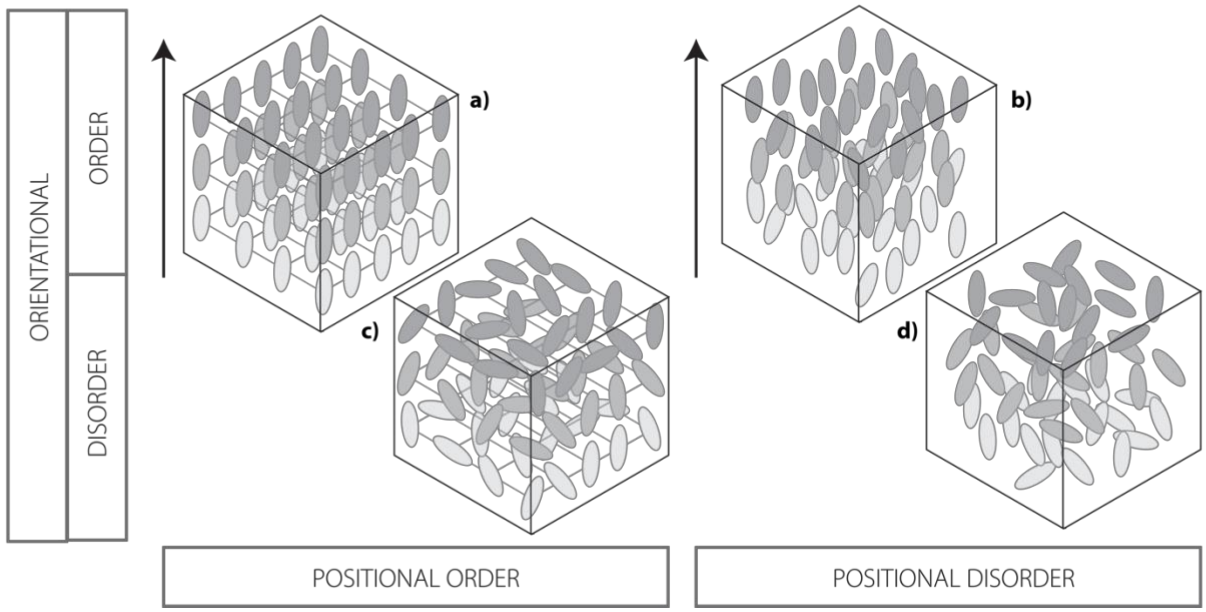

Fig. S1. Schematic illustration of anisotropic particles arranged with different degrees of orientational and positional order. Particles arranged in a crystalline state feature both positional and orientational order (a). A nematic liquid crystal possesses orientational order, but particles are randomly positioned (b). If particles are arranged in a positionally ordered structure, but have random orientation, they constitute a glassy crystal (c). The isotropic liquid phase is reached when both positional and orientational orders are lost (d).

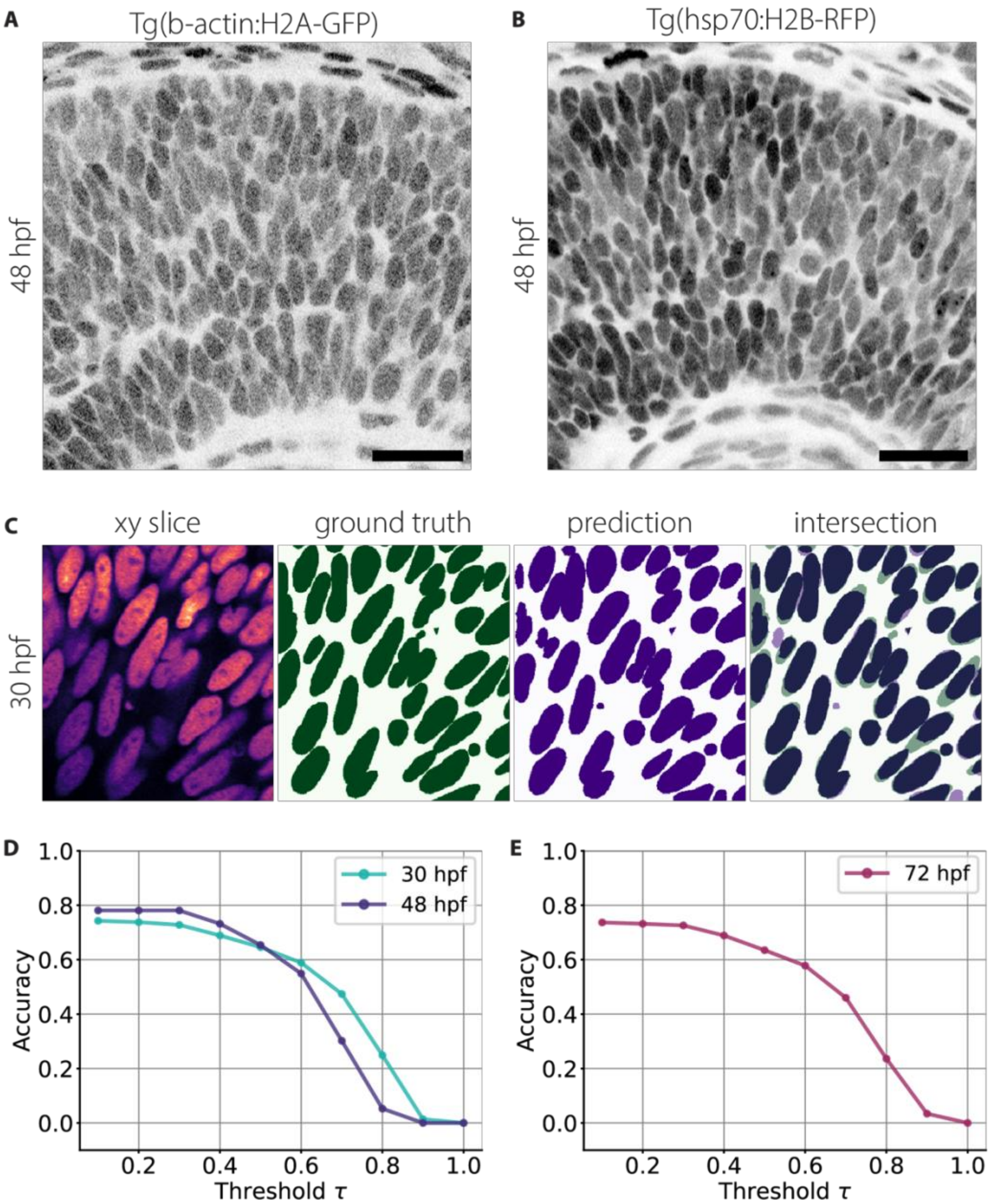

Fig. S2. **Training and evaluation of custom StarDist-3D models. A), B)** Representative confocal sections of 48 hpf retinas from Tg(hsp70:H2B-RFP) in panel (A) and Tg(b-act:H2A-GFP) embryos in panel (B). Scale bar is 20 µm. **C)** Representative optical slices of one cropped stack from the test dataset. Corresponding manually annotated ground truth image and StarDist-3D prediction are shown, together with their overlap. **D)** Accuracy for several IoU thresholds for evaluation datasets from 30 hpf and 48 hpf retinas for StarDist-3D model A. The number of test ground-truth stacks per stage is: 30 hpf, N = 2 images, n = 272 nuclei; 48 hpf, N = 3; n = 225. **E)** Accuracy for several IoU thresholds for evaluation datasets from 72 hpf retinas for StarDist-3D model B. 72 hpf, N images = 4, n nuclei = 647.

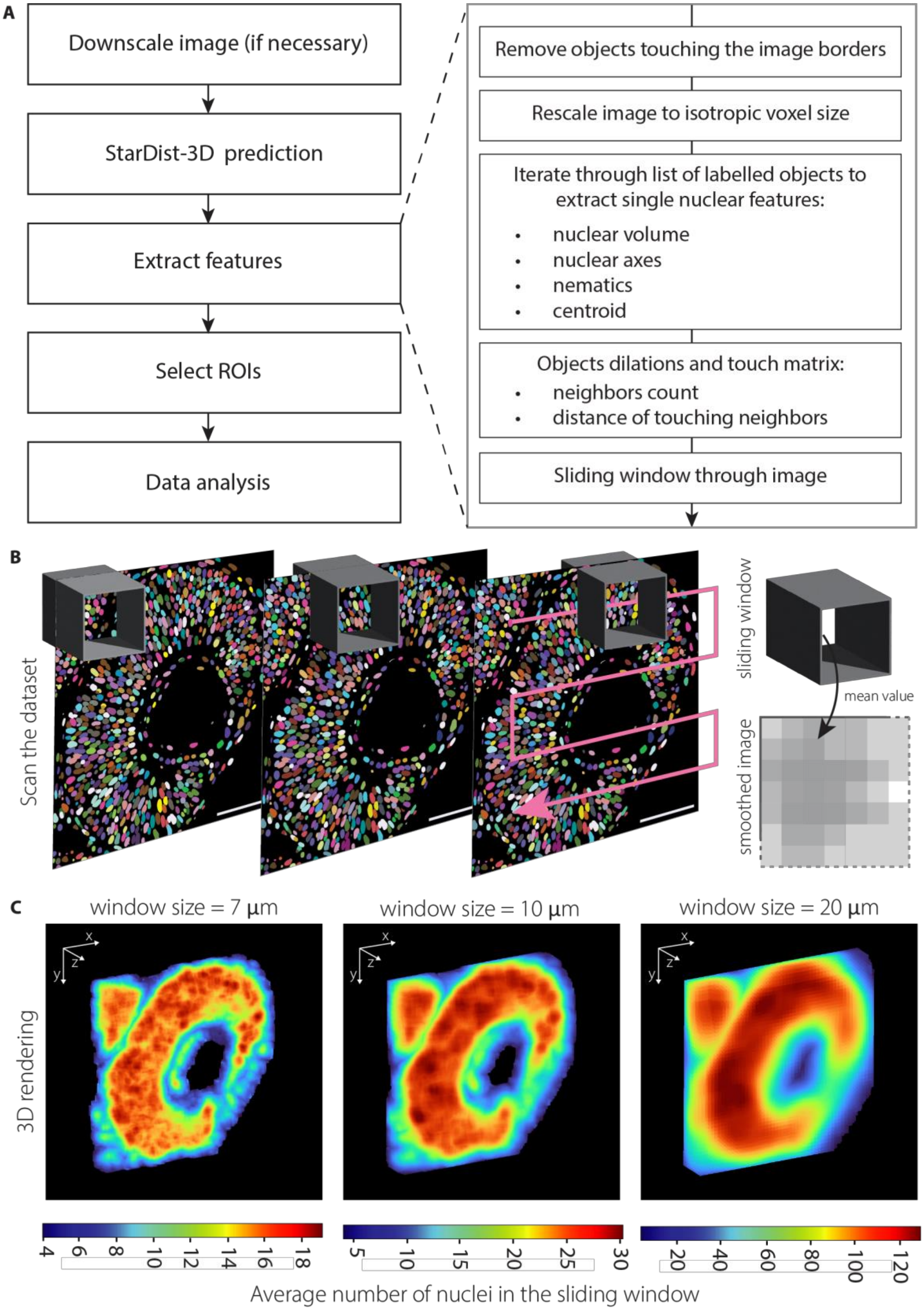

Fig. S3. Analysis pipeline of volumetric imaging dataset. A) Workflow of the established 3D

image analysis pipeline. **B)** Schematics showing the procedure to smooth volumetric imaging dataset. The cubic window iterated through the image stack of a 42 hpf segmented retina and the average value for the feature of interest was calculated and assigned to the pixels inside the window. Ultimately, a smoothed dataset reporting average values computed for each pixel is generated. Scale bar is 50 μm. **C)** Representative 3D renderings of the 42 hpf segmented retina shown in panel (B) after smoothing the number of nuclei within different window sizes. The pixel intensities correspond to the average number of nuclei within the window.

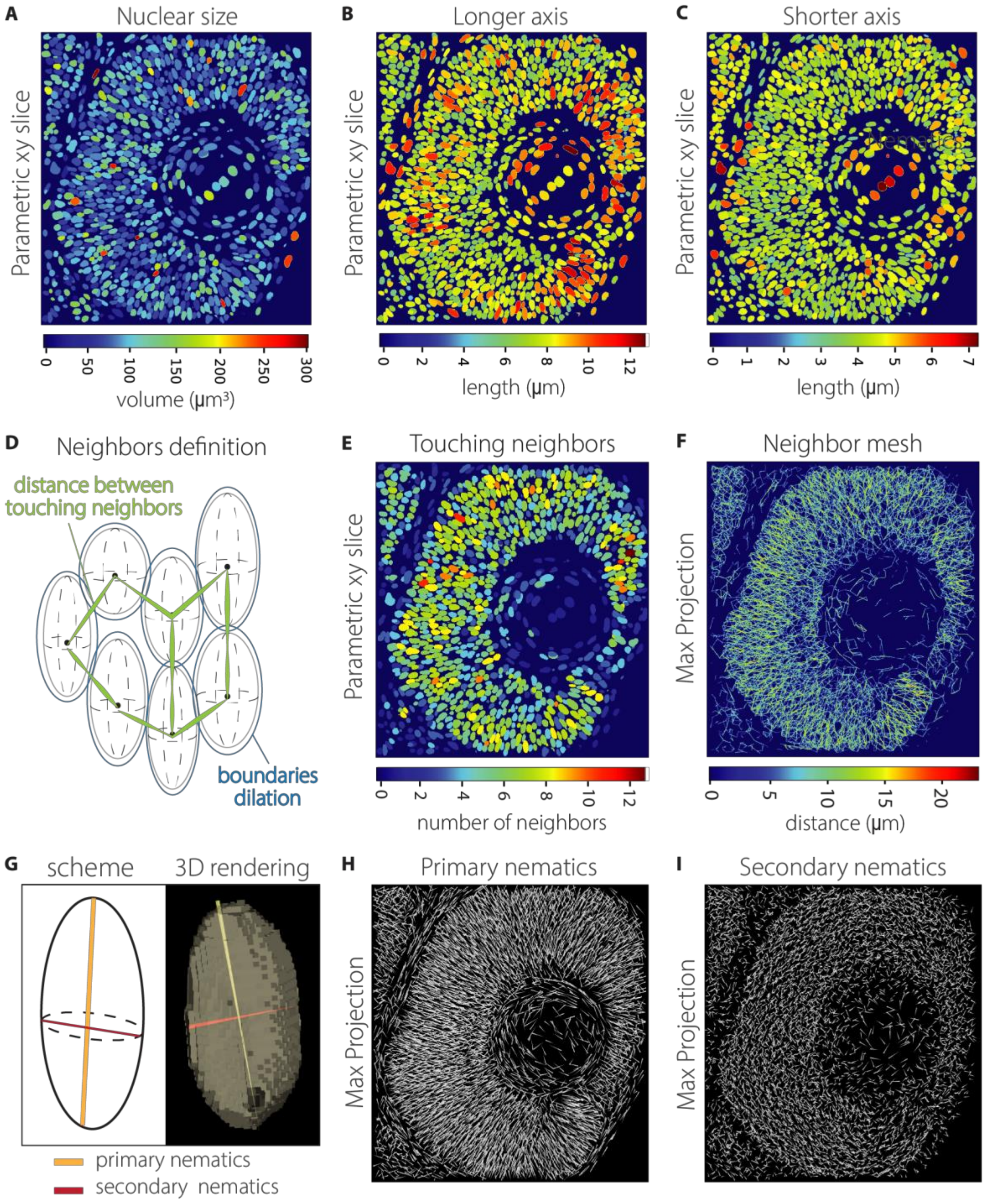

Fig. S4. Extraction of nuclear shape and neighbourhood descriptors. A), B), C), E) Representatives optical slices of parametric images of a 42 hpf segmented retina. Parametric images showing volume of nuclei (A), lengths of the longer (B) and shorter (C) axes and number of touching neighbours (E) in a 42 hpf retina. **D)** Schematic illustration showing the definition of touching neighbours. Boundaries of the nuclei were expanded for a given radius (dilated nuclei outlined in blue) and the mesh of internuclear distances between touching neighbours was computed (in green). **F)** Maximum intensity projection of distance mesh between touching nuclei

from panel (D) and (E) in a 42 hpf retina. **G)** Schematic illustration of primary and secondary nematic axes, together with the 3D rendering of a nucleus showing its primary and secondary nematics. **H), I)** Maximum intensity projections of primary (H) and secondary (I) nematics in a 42 hpf retina.

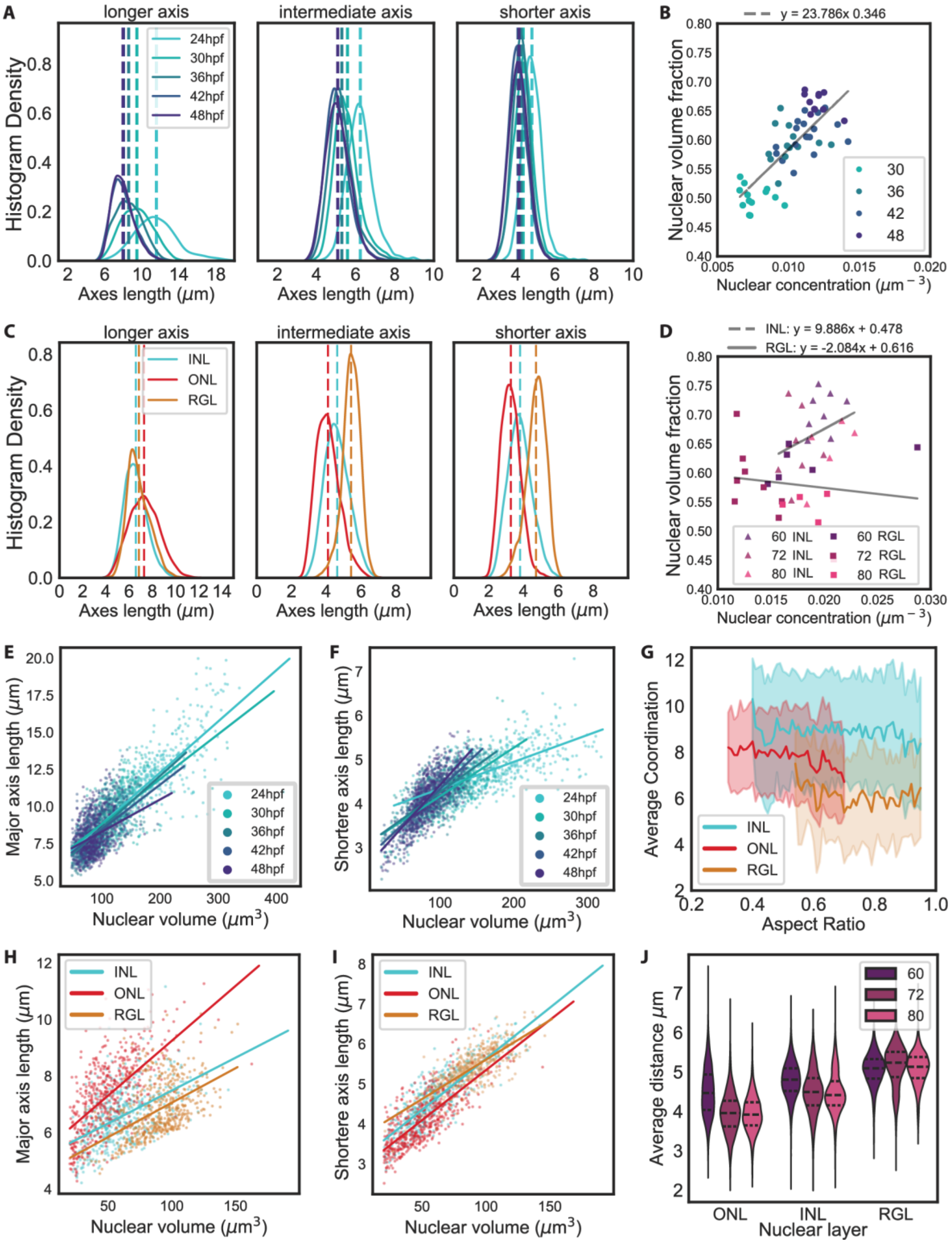

Fig. S5. Nuclear shapes and positioning change during the transition from a PSE to a laminated retina. A), C) Histogram density distributions of axis lengths of nuclei in the RNE during the proliferative phase (A) and in 72 hpf retinas (C). Color-coded and segmented lines in panels (A, C) indicate the mean values for each distribution. **B), D)** Scatter plots showing linear correlation between nuclear volume fractions and nuclear concentrations between 30 hpf and 48

hpf (B) and between 60 hpf and 80 hpf (D). **E), F)** Scatter plots between nuclear volumes and longer axis lengths (E) or shorter axis lengths (F) during the proliferative phase of the RNE. Pearson's correlation coefficient for panel (E): 24 hpf, r = 0.79; 30 hpf, r = 0.65; 36 hpf, r = 0.66; 42 hpf, r = 0.56; 48 hpf, r = 0.36. Pearson's correlation coefficient for panel (F): 24 hpf, r = 0.64; 30 hpf, r = 0.72; 36 hpf, r = 0.71; 42 hpf, r = 0.75; 48 hpf, r = 0.75. **G)** Correlation between average coordinations numbers and nuclear aspect ratios in 72 hpf retinas across the three nuclear layers. Solid lines show mean values, shaded area indicates the standard deviation from the mean. **H), I)** Scatter plots between nuclear volumes and longer axis lengths (H) or shorter axis lengths (I) in 72 hpf retinas. Pearson's correlation coefficient for panel (H): ONL, r = 0.00; INL, r = 0.00; RGL, r = 0.00. Pearson's correlation coefficient for panel (I): ONL, r = 0.00; INL, r = 0.00; RGL, r = 0.00. **J)** Distributions of average distances across the three nuclear layers between 60 and 80 hpf.

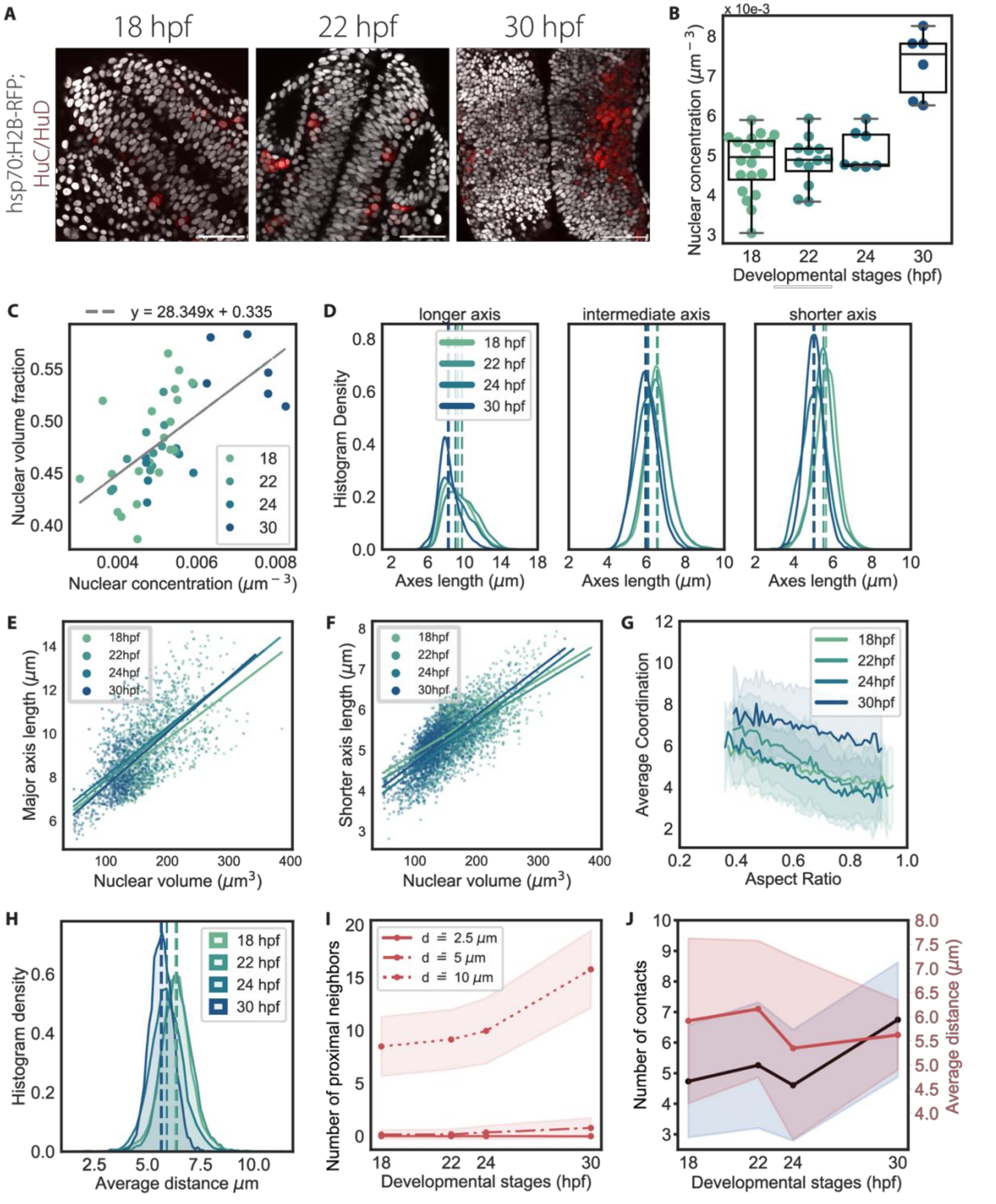

Fig. S6. Nuclear shape descriptors to follow nuclear packing changes in the hindbrain. A) Neuronal differentiation starts around 18 hpf in the hindbrain. Huc/HuD-positive neurons are marked in red. Nuclei are labelled with Tg(hsp70:H2B-RFP). Scale bar is 50 μm. **B)** Concentration of nuclei found within the ROIs of Fig. 3 C. **C)** Scatter plot showing linear correlation between nuclear volume fractions and concentrations during hindbrain development between 18 and 30

hpf. **D)** Histogram density distributions of nuclear axis lengths over development. Segmented lines indicate the mean values for each distribution. **E), F)** Scatter plots between nuclear volumes and longer axis lengths (E) or shorter axis lengths (F) to detect their correlation. Pearson's correlation coefficient (r) for panel (E): 18 hpf, r = 0.61; 22 hpf, r = 0.60; 24 hpf, r = 0.54; 30 hpf, r = 0.66. Pearson's correlation coefficient (r) for panel (F): 18 hpf, r = 0.65; 22 hpf, r = 0.67; 24 hpf, r = 0.69; 30 hpf, r = 0.73. **G)** Correlation between average coordination numbers and nuclear aspect ratios. Solid lines show mean values, shaded area indicate the standard deviation from the mean. **H)** Histogram density distributions of average distances between touching neighbours. **I)** Number of proximal neighbours within different distance radii. **J)** Mean number of contacts within ROIs increase, while the average distance between touching neighbours oscillates around the same value. Solid lines report mean values, shaded area indicate the standard deviation from the mean.

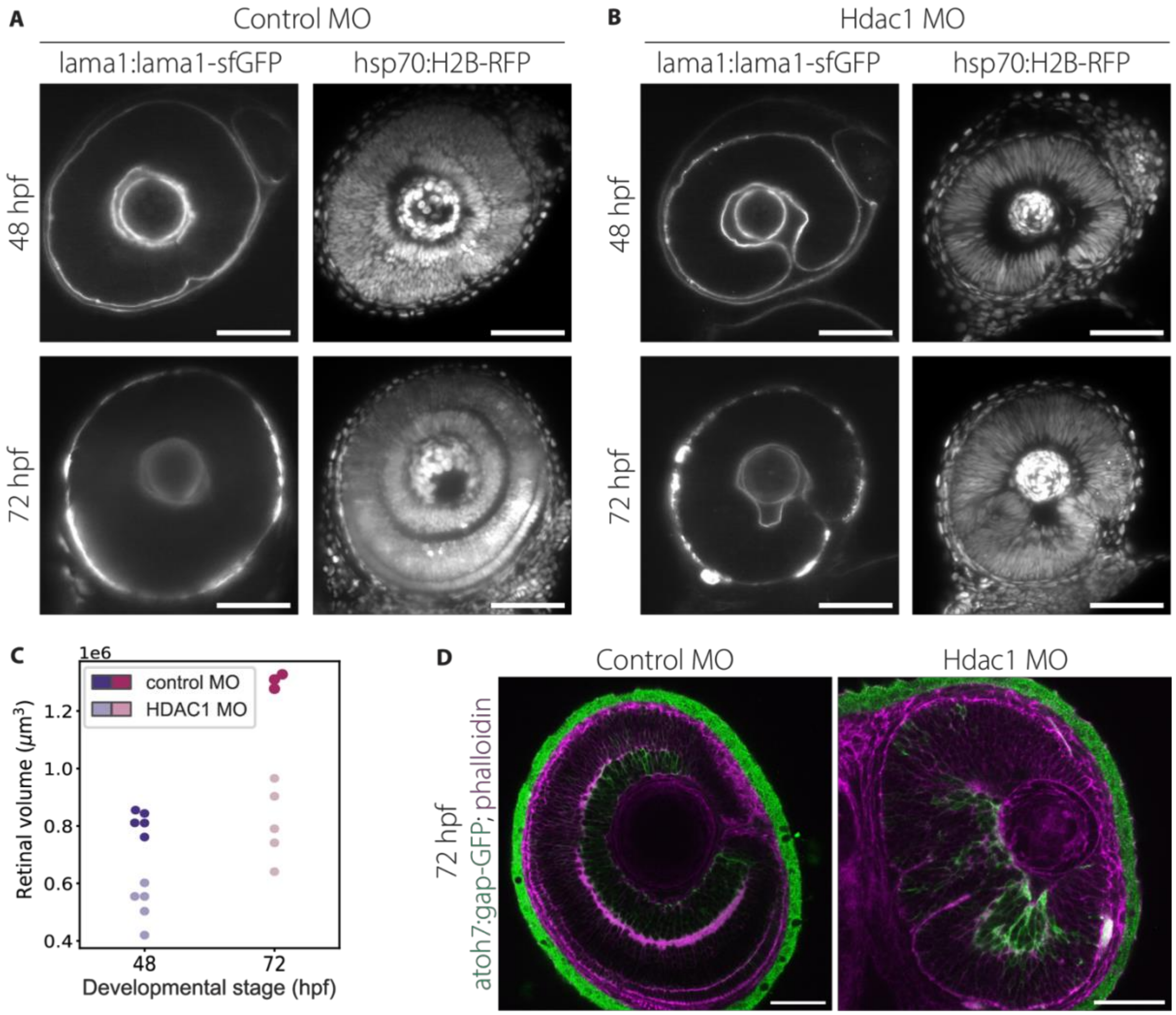

Fig. S7. HDAC1 morphants feature reduced tissue growth and delayed neurogenesis. A), B) Representative optical sections of the eyes of transgenic embryos that were injected either with control morpholino (MO) (panel A) or with HDAC1 MO (panel B). Embryos were imaged at 48 hpf and 72 hpf using the ZEISS Lightsheet Z1. Nuclei are labelled with tg(hsp70:H2B-RFP), the basal lamina is marked with tg(lama1:lama1-sfGFP). Scale bar is 50 µm. **C)** Eye volume quantified from tg(hsp70:H2B-RFP) x tg(hsp70:H2B-RFP) embryos. N = 3 to 5 embryos per condition. **D)** Representative confocal optical sections of the eyes of 72 hpf transgenic embryos injected either with control MO or with HDAC1 MO. The cytoskeleton is labelled with phalloidin (magenta), atoh7-positive neurons are labelled with tg(atoh7:gap-GFP) and signal was enhanced via staining with anti-GFP antibodies (green). Scale bar is 50 µm.

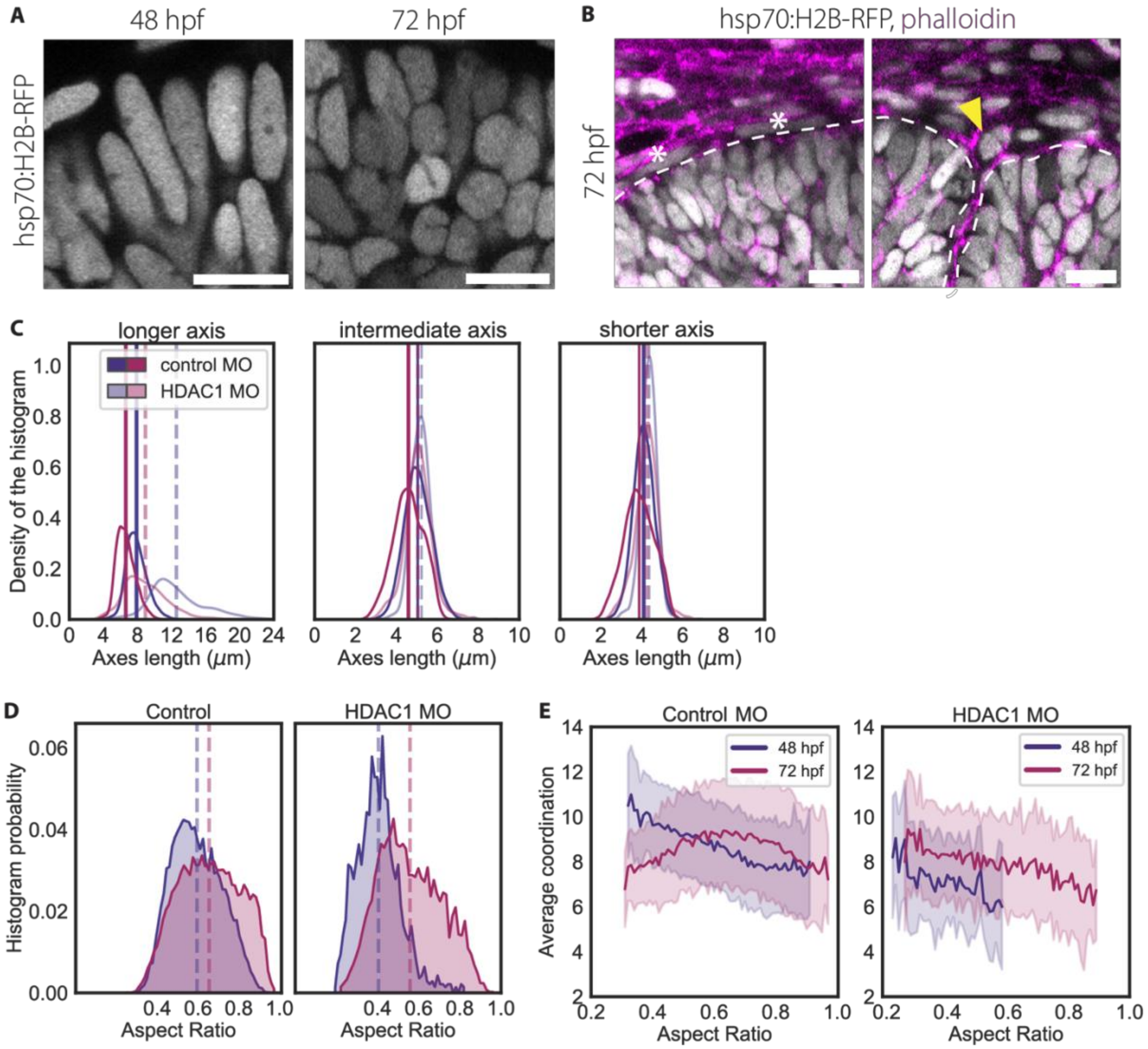

Fig. S8. Nuclei packing densities reach a limit when neurogenesis is delayed in HDAC1 morphants. A) Representative cropped confocal sections of 48 hpf and 72 hpf retinas in HDAC1 morphants. Nuclei are labelled with tg(hsp70:H2B-RFP). Scale bar is 10 µm. **B)** Representative confocal sections of different regions of the retina from one HDAC1 morphant at 72 hpf. The sections show part of the RNE and RPE in regions where the tissue is not buckled (left) and where it is buckled (right). Nuclei of RPE cells appear to be stretched (asterisks) where the tissue is not buckled, while they are more rounded over buckled regions (triangle). Nuclei are labelled with tg(hsp70:H2B-RFP) in grey, the actomyosin skeleton is labelled with phalloidin in magenta. Scale bar is 10 µm. **C)** Histogram density distributions of nuclear axis lengths in control and HDAC1 morphants at 48 hpf and 72 hpf. Color-coded segmented lines indicate the mean values for HDAC1 morphants. Color-coded solid lines indicate the mean values for control morphants. **D)** Histogram density distributions of nuclear aspect ratios, which were defined as the ratio between the longer axis length and the mean length between the intermediate and shorter axes. Color-coded segmented lines indicate the mean values. **E)** Correlation between average coordination numbers and nuclear

aspect ratios in 48 hpf and 72 hpf retinas from control (left) and HDAC1 (right) morphants. Solid lines show mean values, shaded area indicate the standard deviation from the mean.

STARDIST-3D MODEL A

Threshold τ	0.10	0.20	0.30	0.40	0.50	0.60	0.70	0.80	0.90
				30 hpf					
**Accuracy**	0.743	0.738	0.728	0.689	0.646	0.589	0.474	0.249	0.0137
**Precision**	0.789	0.787	0.781	0.755	0.727	0.687	0.596	0.369	0.025
**Recall**	0.926	0.922	0.915	0.886	0.853	0.805	0.699	0.434	0.029
				48 hpf					
**Accuracy**	0.781	0.781	0.781	0.732	0.654	0.549	0.302	0.0526	0.000
**Precision**	0.898	0.898	0.898	0.865	0.809	0.726	0.474	0.102	0.000
**Recall**	0.858	0.858	0.858	0.827	0.773	0.693	0.453	0.098	0.000

STARDIST-3D MODEL B

Threshold τ	0.10	0.20	0.30	0.40	0.50	0.60	0.70	0.80	0.90
				72 hpf					
**Accuracy**	0.737	0.732	0.726	0.689	0.635	0.578	0.460	0.236	0.034
**Precision**	0.945	0.941	0.937	0.909	0.865	0.816	0.702	0.425	0.072
**Recall**	0.769	0.767	0.764	0.740	0.705	0.665	0.571	0.346	0.059

Table S1. Evaluation of StarDist-3D models. Accuracy, precision and recall scores for several Intersection over Union (IoU) thresholds $\boldsymbol{\tau}$ for test datasets from embryos stages at 30 hours post fertilization (hpf) and 48 hpf (model A) and 72 hpf (model B).

Movie S1. 3D rendering of segmented nuclei and corresponding parametric images showing some of the extracted nuclear measurements. Representative volumetric rendering of nuclei after segmentation using our trained StarDist-3D model and corresponding parametric images where parameter values with color coding were used as labels for each nucleus. Parametric values shown are the nuclear volume, the length of long and short nuclear axes, the count of touching neighbors and the count of proximal neighbors at a distance of 50 pixels. Parametric values are color coded using the turbo colormap from the python package matplotlib, where lower values are shown in blue and higher values in red. 3D rendering was produced using the animation plugin in Napari. Nuclei belong to a 30 hpf zebrafish embryo.

Movie S2. 3D rendering of segmented nuclei and their nematic axes. Representative volumetric rendering of nuclei after segmentation using our trained StarDist-3D model and visualization of their primary and secondary nematic axes. Nuclei belong to the same 30 hpf zebrafish embryo shown in Movie S1.

Movie S3. 3D rendering of segmented nuclei from the selected region of interest (ROI). Representative volumetric rendering of nuclei segmented using our trained StarDist-3D model. The ROI was selected in the central portion of the retinal PSE, using the lens to determine the depth, as explained in Materials and Methods. Nuclei belong to the same 30 hpf zebrafish embryo shown in Movie S1.

REFERENCES AND NOTES

1. I. H. Smart, Proliferative characteristics of the ependymal layer during the early development of the spinal cord in the mouse. (1972).

2. T. Miyata, Development of three-dimensional architecture of the neuroepithelium: Role of pseudostratification and cellular 'community'. *Dev. Growth Differ.* **50**, S105–S112 (2008).

3. M. A. Ferreira, E. Despin-Guitard, F. Duarte, P. Degond, E. Theveneau, Interkinetic nuclear movements promote apical expansion in pseudostratified epithelia at the expense of apicobasal elongation. *PLoS Comput. Biol.* **15**, e1007171 (2019).

4. P. Guerrero, R. Perez-Carrasco, M. Zagorski, D. Page, A. Kicheva, J. Briscoe, K. M. Page, Neuronal differentiation influences progenitor arrangement in the vertebrate neuroepithelium. *Development* **146**, dev176297 (2019).

5. A. Kicheva, T. Bollenbach, A. Ribeiro, H. Perez Valle, R. Lovell-Badge, V. Episkopou, J. Briscoe, Coordination of progenitor specification and growth in mouse and chick spinal cord. *Science* **345**, 1254927 (2014).

6. A. Iulianella, M. Sharma, M. Durnin, G. B. Vanden Heuvel, P. A. Trainor, *Cux2* (*Cutl2*) integrates neural progenitor development with cell-cycle progression during spinal cord neurogenesis. *Development* **135**, 729–741 (2008).

7. I. Bystron, C. Blakemore, P. Rakic, Development of the human cerebral cortex: Boulder Committee revisited. *Nat. Rev. Neurosci.* **9**, 110–122 (2008).

8. M. A. Lancaster, M. Renner, C.-A. Martin, D. Wenzel, L. S. Bicknell, M. E. Hurles, T. Homfray, J. M. Penninger, A. P. Jackson, J. A. Knoblich, Cerebral organoids model human brain development and microcephaly. *Nature* **501**, 373–379 (2013).

9. M. Eiraku, N. Takata, H. Ishibashi, M. Kawada, E. Sakakura, S. Okuda, K. Sekiguchi, T. Adachi, Y. Sasai, Self-organizing optic-cup morphogenesis in three-dimensional culture. *Nature* **472**, 51–56 (2011).

10. T. Nakano, S. Ando, N. Takata, M. Kawada, K. Muguruma, K. Sekiguchi, K. Saito, S. Yonemura, M. Eiraku, Y. Sasai, Self-formation of optic cups and storable stratified neural retina from human ESCs. *Cell Stem Cell* **10**, 771–785 (2012).

11. R. Bort, M. Signore, K. Tremblay, J. P. M. Barbera, K. S. Zaret, Hex homeobox gene controls the transition of the endoderm to a pseudostratified, cell emergent epithelium for liver bud development. *Dev. Biol.* **290**, 44–56 (2006).

12. E. J. Meyer, A. Ikmi, M. C. Gibson, Interkinetic nuclear migration is a broadly conserved feature of cell division in pseudostratified epithelia. *Curr. Biol.* **21**, 485–491 (2011).

13. C. Norden, Pseudostratified epithelia – cell biology, diversity and roles in organ formation at a glance. *J. Cell Sci.* **130**, 1859–1863 (2017).

14. P. J. Strzyz, M. Matejcic, C. Norden, *Heterogeneity, Cell Biology and Tissue Mechanics of Pseudostratified Epithelia: Coordination of Cell Divisions and Growth in Tightly Packed Tissues* (Elsevier Inc., 2016; http://dx.doi.org/10.1016/bs.ircmb.2016.02.004)vol. **325**.

15. H. F. Gómez, M. S. Dumond, L. Hodel, R. Vetter, D. Iber, 3D cell neighbour dynamics in growing pseudostratified epithelia. *eLife* **10**, e68135 (2021).

16. G. C. Schoenwolf, M. L. Powers, Shaping of the chick neuroepithelium during primary and secondary neurulation: Role of cell elongation. *Anat. Rec.* **218**, 182–195 (1987).

17. F. C. Sauer, Mitosis in the neural tube. *J. Comp. Neurol.* **62**, 377–405 (1935).

18. L. Leung, A. V. Klopper, S. W. Grill, W. A. Harris, C. Norden, Apical migration of nuclei during G2 is a prerequisite for all nuclear motion in zebrafish neuroepithelia. *Development* **139**, 2635–2635 (2012).

19. C. Norden, S. Young, B. A. Link, W. A. Harris, Actomyosin Is the Main Driver of Interkinetic Nuclear Migration in the Retina. *Cell* **138**, 1195–1208 (2009).

20. A. Azizi, A. Herrmann, Y. Wan, S. J. R. P. Buse, P. J. Keller, R. E. Goldstein, W. A. Harris, Nuclear crowding and nonlinear diffusion during interkinetic nuclear migration in the zebrafish retina. *eLife* **9**, e58635 (2020).

21. M. Matejčić, G. Salbreux, C. Norden, A non-cell-autonomous actin redistribution enables isotropic retinal growth. *PLoS Biol.* **16**, e2006018 (2018).

22. P. J. Strzyz, H. O. Lee, J. Sidhaye, I. P. Weber, L. C. Leung, C. Norden, Interkinetic Nuclear Migration Is Centrosome Independent and Ensures Apical Cell Division to Maintain Tissue Integrity. *Dev. Cell* **32**, 203–219 (2015).

23. X. Morin, F. Jaouen, P. Durbec, Control of planar divisions by the G-protein regulator LGN maintains progenitors in the chick neuroepithelium. *Nat. Neurosci.* **10**, 1440–1448 (2007).

24. Y. Nakajima, E. J. Meyer, A. Kroesen, S. A. McKinney, M. C. Gibson, Epithelial junctions maintain tissue architecture by directing planar spindle orientation. *Nature* **500**, 359–362 (2013).

25. J. A. Adelmann, R. Vetter, D. Iber, The impact of cell size on morphogen gradient precision. *Development* **150**, dev201702 (2023).

26. L. Bocanegra-Moreno, A. Singh, E. Hannezo, M. Zagorski, A. Kicheva, Cell cycle dynamics control fluidity of the developing mouse neuroepithelium. *Nat. Phys.* **19**, 1050–1058. (2023).

27. F. Guilak, J. R. Tedrow, R. Burgkart, Viscoelastic properties of the cell nucleus. *Biochem. Biophys. Res. Commun.* **269**, 781–786 (2000).

28. J. Lammerding, “Mechanics of the Nucleus” in Comprehensive Physiology, Y. S. Prakash, Ed. (Wiley, ed. 1, 2011; https://onlinelibrary.wiley.com/doi/10.1002/cphy.c100038), pp. 783–807.

29. A. L. McGregor, C.-R. Hsia, J. Lammerding, Squish and squeeze — the nucleus as a physical barrier during migration in confined environments. *Curr. Opin. Cell Biol.* **40**, 32–40 (2016).

30. Y. Kalukula, A. D. Stephens, J. Lammerding, S. Gabriele, Mechanics and functional consequences of nuclear deformations. *Nat. Rev. Mol. Cell Biol.* **23**, 583–602 (2022).

31. M. Ishii, T. Tateya, M. Matsuda, T. Hirashima, Stalling interkinetic nuclear migration in curved pseudostratified epithelium of developing cochlea. *R. Soc. Open Sci.* **8**, 211024 (2021).

32. S. Kim, R. Amini, S.-T. Yen, P. Pospíšil, A. Boutillon, I. A. Deniz, O. Campàs, A nuclear jamming transition in vertebrate organogenesis. *Nat. Mater.* **23**, 1592–1599 (2024).

33. P. Gómez-Gálvez, P. Vicente-Munuera, A. Tagua, C. Forja, A. M. Castro, M. Letrán, A. Valencia-Expósito, C. Grima, M. Bermúdez-Gallardo, Ó. Serrano-Pérez-Higueras, F. Cavodeassi, S. Sotillos, M. D. Martín-Bermudo, A. Márquez, J. Buceta, L. M. Escudero, Scutoids are a geometrical solution to three-dimensional packing of epithelia. *Nat. Commun.* **9**, 2960 (2018).

34. M. Weigert, U. Schmidt, R. Haase, K. Sugawara, G. Myers, Star-convex polyhedra for 3D object detection and segmentation in microscopy. arXiv:1908.03636 [cs.CV] (2019).

35. E. Nerli, M. Rocha-Martins, C. Norden, Asymmetric neurogenic commitment of retinal progenitors involves notch through the endocytic pathway. *eLife* **9**, e60462 (2020).

36. J. Icha, C. Kunath, M. Rocha-Martins, C. Norden, Independent modes of ganglion cell translocation ensure correct lamination of the zebrafish retina. *J. Cell Biol.* **215**, 259–275 (2016).

37. M. Rocha-Martins, E. Nerli, J. Kretzschmar, M. Weigert, J. Icha, E. W. Myers, C. Norden, Neuronal migration prevents spatial competition in retinal morphogenesis. *Nature* **620**, 615–624 (2023).

38. R. Amini, A. Bhatnagar, C. Norden, C. Bioengineering, Amoeboid-like neuronal migration ensures correct horizontal cell layer formation in the developing vertebrate retina, *eLife* **11**, e76408 (2021).

39. C. Norden, A fish eye view: Retinal morphogenesis from optic cup to neuronal lamination. *Annu. Rev. Cell Dev. Biol.* **39**, 175–196 (2023).

40. R. Amini, M. Rocha-Martins, C. Norden, Neuronal migration and lamination in the vertebrate retina. *Front. Neurosci.* **11**, 742 (2018).

41. D.-E. Nilsson, The evolution of eyes and visually guided behaviour. *Philos. Trans. R. Soc. B* **364**, 2833–2847 (2009).

42. I. Y. Buchsbaum, S. Cappello, Neuronal migration in the CNS during development and disease: Insights from in vivo and in vitro models. *Development* **146**, dev163766 (2019).

43. J. G. Gleeson, C. A. Walsh, Neuronal migration disorders: From genetic diseases to developmental mechanisms. *Trends Neurosci.* **23**, 352–359 (2000).

44. C. Cepko, Intrinsically different retinal progenitor cells produce specific types of progeny. *Nat. Rev. Neurosci.* **15**, 615–627 (2014).

45. E. M. Levine, E. S. Green, Cell-intrinsic regulators of proliferation in vertebrate retinal progenitors. *Semin. Cell Dev. Biol.* **15**, 63–74 (2004).

46. P. M. Chaikin, T. C. Lubensky, *Principles of Condensed Matter Physics.* (Cambridge Univ. Press, 2012).

47. A. Doostmohammadi, B. Ladoux, Physics of liquid crystals in cell biology. *Trends Cell Biol.* **32**, 140–150 (2022).

48. G. D. Scott, D. M. Kilgour, The density of random close packing of spheres. *J. Phys. D Appl. Phys.* **2**, 863–866 (1969).

49. J. G. Berryman, Random close packing of hard spheres and disks. *Phys. Rev. A* **27**, 1053–1061 (1983).

50. A. Donev, I. Cisse, D. Sachs, E. A. Variano, F. H. Stillinger, R. Connelly, S. Torquato, P. M. Chaikin, Improving the Density of Jammed Disordered Packings Using Ellipsoids. *Science* **303**, 990–993 (2004).

51. S. Torquato, T. M. Truskett, P. G. Debenedetti, Is Random Close Packing of Spheres Well Defined? *Phys. Rev. Lett.* **84**, 2064–2067 (2000).

52. M. Maia-Gil, M. Gorjão, R. Belousov, J. A. Espina, J. Coelho, A. P. Ramos, E. H. Barriga, A. Erzberger, C. Norden, Nuclear deformability facilitates apical nuclear migration in the developing zebrafish retina. bioRxiv 588091[Preprint] (2024); https://doi.org/10.1101/2024.04.04.588091.

53. I. Yanakieva, A. Erzberger, M. Matejčić, C. D. Modes, C. Norden, Cell and tissue morphology determine actin-dependent nuclear migration mechanisms in neuroepithelia. *J. Cell Biol.* **218**, 3272–3289 (2019).

54. C. F. Hevia, C. Engel-Pizcueta, F. Udina, C. Pujades, The neurogenic fate of the hindbrain boundaries relies on Notch3-dependent asymmetric cell divisions. *Cell Rep.* **39**, 110915 (2022).

55. B. Ciruna, A. Jenny, D. Lee, M. Mlodzik, A. F. Schier, Planar cell polarity signalling couples cell division and morphogenesis during neurulation. *Nature* **439**, 220–224 (2006).

56. E. Hong, R. Brewster, *N-cadherin* is required for the polarized cell behaviors that drive neurulation in the zebrafish. *Development* **133**, 3895–3905 (2006).

57. G. Salbreux, L. K. Barthel, P. A. Raymond, D. K. Lubensky, Coupling mechanical deformations and planar cell polarity to create regular patterns in the zebrafish retina. *PLoS Comput. Biol.* **8**, e1002618 (2012).

58. A. Trushko, I. Di Meglio, A. Merzouki, C. Blanch-Mercader, S. Abuhattum, J. Guck, K. Alessandri, P. Nassoy, K. Kruse, B. Chopard, A. Roux, Buckling of an epithelium growing under spherical confinement. *Dev. Cell* **54**, 655–668.e6 (2020).

59. R. Amini, A. Bhatnagar, R. Schlüßler, S. Möllmert, J. Guck, C. Norden, Amoeboid-like migration ensures correct horizontal cell layer formation in the developing vertebrate retina. *eLife* **11**, e76408 (2022).

60. J. A. Stadler, A. Shkumatava, W. H. J. Norton, M. J. Rau, R. Geisler, S. Fischer, C. J. Neumann, Histone deacetylase 1 is required for cell cycle exit and differentiation in the zebrafish retina. *Dev. Dyn.* **233**, 883–889 (2005).

61. M. Yamaguchi, N. Tonou-Fujimori, A. Komori, R. Maeda, Y. Nojima, H. Li, H. Okamoto, I. Masai, Histone deacetylase 1 regulates retinal neurogenesis in zebrafish by suppressing Wnt ad Notch signaling pathways. *Development* **132**, 3027–3043 (2005).

62. V. T. Cunliffe, Histone deacetylase 1 is required to repress Notch target gene expression during zebrafish neurogenesis and to maintain the production of motoneurones in response to hedgehog signalling. *Development* **131**, 2983–2995 (2004).

63. D. Iber, R. Vetter, Relationship between epithelial organization and morphogen interpretation. *Curr. Opin. Genet. Dev.* **75**, 101916 (2022).

64. N. F. De Leeuw, R. Budhathoki, L. J. Russell, D. Loerke, J. T. Blankenship, Nuclei as mechanical bumpers during epithelial remodeling. *J. Cell Biol.* **223**, e202405078 (2024).

65. A. Reichenbach, S. Agte, M. Francke, K. Franze, How light traverses the inverted vertebrate retina: No flaw of nature. *e-Neuroforum* **5**, 93–100 (2014).

66. K. Franze, J. Grosche, S. N. Skatchkov, S. Schinkinger, C. Foja, D. Schild, O. Uckermann, K. Travis, A. Reichenbach, J. Guck, Müller cells are living optical fibers in the vertebrate retina. *Proc. Natl. Acad. Sci. U.S.A.* **104**, 8287–8292 (2007).

67. I. Solovei, M. Kreysing, C. Lanctôt, S. Kösem, L. Peichl, T. Cremer, J. Guck, B. Joffe, Nuclear architecture of rod photoreceptor cells adapts to vision in mammalian evolution. *Cell* **137**, 356–368 (2009).

68. M. Kreysing, L. Boyde, J. Guck, K. J. Chalut, Physical insight into light scattering by photoreceptor cell nuclei. *Opt. Lett.* **35**, 2639–2641 (2010).

69. E. Hannezo, J. Prost, J.-F. Joanny, Theory of epithelial sheet morphology in three dimensions. *Proc. Natl. Acad. Sci. U.S.A.* **111**, 27–32 (2014).

70. D. Drasdo, Buckling instabilities of one-layered growing tissues. *Phys. Rev. Lett.* **84**, 4244–4247 (2000).

71. C. B. Kimmel, W. W. Ballard, S. R. Kimmel, B. Ullmann, T. F. Schilling, Stages of embryonic development of the zebrafish. *Dev. Dyn.* **203**, 253–310 (1995).

72. B. Geldmacher-Voss, A. M. Reugels, S. Pauls, J. A. Campos-Ortega, A 90° rotation of the mitotic spindle changes the orientation of mitoses of zebrafish neuroepithelial cells. *Development* **130**, 3767–3780 (2003).

73. E. Dzafic, P. J. Strzyz, M. Wilsch-Bräuninger, C. Norden, Centriole amplification in zebrafish affects proliferation and survival but not differentiation of neural progenitor cells. *Cell Rep.* **13**, 168–182 (2015).

74. F. R. Zolessi, L. Poggi, C. J. Wilkinson, C.-B. Chien, W. A. Harris, Polarization and orientation of retinal ganglion cells in vivo. *Neural Dev.* **1**, 2 (2006).

75. K. G. Soans, A. P. Ramos, J. Sidhaye, A. Krishna, A. Solomatina, K. B. Hoffmann, R. Schlüßler, J. Guck, I. F. Sbalzarini, C. D. Modes, C. Norden, Collective cell migration during optic cup formation features changing cell-matrix interactions linked to matrix topology. *Curr. Biol.* **32**, 4817–4831.e9 (2022).

76. J. Schindelin, I. Arganda-Carreras, E. Frise, V. Kaynig, M. Longair, T. Pietzsch, S. Preibisch, C. Rueden, S. Saalfeld, B. Schmid, J.-Y. Tinevez, D. J. White, V. Hartenstein, K. Eliceiri, P. Tomancak, A. Cardona, Fiji: An open-source platform for biological-image analysis. *Nat. Methods* **9**, 676–682 (2012).

77. S. Machado, V. Mercier, N. Chiaruttini, LimeSeg: A coarse-grained lipid membrane simulation for 3D image segmentation. *BMC Bioinformatics* **20**, 2 (2019).

78. N. Sofroniew, T. Lambert, G. Bokota, J. Nunez-Iglesias, P. Sobolewski, A. Sweet, L. Gaifas, K. Evans, A. Burt, D. Doncila Pop, K. Yamauchi, M. Weber Mendonça, G. Buckley, W.-M. Vierdag, L. Royer, A. Can Solak, K. I. S. Harrington, J. Ahlers, D. Althviz Moré, O. Amsalem, A. Anderson, A. Annex, P. Boone, J. Bragantini, M. Bussonnier, C. Caporal, J. Eglinger, A. Eisenbarth, J. Freeman, C. Gohlke, K. Gunalan, H. Har-Gil, M. Harfouche, V. Hilsenstein, K. Hutchings, J. Lauer, G. Lichtner, Z. Liu, L. Liu, A. Lowe, L. Marconato, S. Martin, A.

McGovern, L. Migas, N. Miller, H. Muñoz, J.-H. Müller, C. Nauroth-Kreß, D. Palecek, C. Pape, E. Perlman, K. Pevey, G. Peña-Castellanos, A. Pierré, D. Pinto, J. Rodríguez-Guerra, D. Ross, C. T. Russell, J. Ryan, G. Selzer, M. Smith, P. Smith, K. Sofiiuk, J. Soltwedel, D. Stansby, J. Vanaret, P. Wadhwa, M. Weigert, J. Windhager, P. Winston, R. Zhao, napari: A multi-dimensional image viewer for Python, version v0.5.4, (Zenodo. 2024); https://doi.org/10.5281/ZENODO.3555620.

79. C. R. Harris, K. J. Millman, S. J. Van Der Walt, R. Gommers, P. Virtanen, D. Cournapeau, E. Wieser, J. Taylor, S. Berg, N. J. Smith, R. Kern, M. Picus, S. Hoyer, M. H. Van Kerkwijk, M. Brett, A. Haldane, J. F. Del Río, M. Wiebe, P. Peterson, P. Gérard-Marchant, K. Sheppard, T. Reddy, W. Weckesser, H. Abbasi, C. Gohlke, T. E. Oliphant, Array programming with NumPy. *Nature* **585**, 357–362 (2020).

80. S. Van Der Walt, J. L. Schönberger, J. Nunez-Iglesias, F. Boulogne, J. D. Warner, N. Yager, E. Gouillart, T. Yu, scikit-image contributors, scikit-image: Image processing in Python. *PeerJ.* **2**, e453 (2014).

81. S. Rigaud, R. Haase, J. Soltwedel, grahamross123, Marvin Albert, Pradeep Rajasekhar, clEsperanto/pyclesperanto: 0.16.0, version 0.16.0 (Zenodo, 2024); https://doi.org/10.5281/ZENODO.13853800.

82. R. Haase, P. Rajasekhar, Talley Lambert, Grahamross123, J. Nunez-Iglesias, Lachie, C. Caporal, C. Avenel, ENicolay, E. Sandaltzopoulou, clEsperanto/pyclesperanto_prototype: 0.24.1, version 0.24.1 (Zenodo, 2023); https://doi.org/10.5281/ZENODO.7827755.

83. G. Napoli, S. Turzi, Snap buckling of a confined thin elastic sheet. *Proc. R. Soc. A* **471**, 20150444, (2015).

84. A.E.H. Love, *A Treatise on the Mathematical Theory of Elasticity*, (Cambridge Univ. Press, ed. 4, (1927).
